# A Comparison of Semilandmarking Approaches in the Analysis of Size and Shape

**DOI:** 10.3390/ani13071179

**Published:** 2023-03-28

**Authors:** Wuyang Shui, Antonio Profico, Paul O’Higgins

**Affiliations:** 1 Department of Archaeology, University of York, King’s Manor, York YO1 7EP, UK; 2 Department of Biology, University of Pisa, Via Derna 1, 56126 Pisa, Italy; 3 Hull York Medical School, University of York, York YO10 5DD, UK

**Keywords:** geometric morphometrics, virtual anthropology, homology, size and shape variation, semilandmarking methods, semilandmark densities

## Abstract

**Simple Summary:**

Landmarks are commonly used to investigate how objects vary in form. However, many objects present few identifiable landmarks. To remedy this, several approaches have been developed to densely match points between surfaces lacking readily identifiable landmarks. These matched points are termed semilandmarks. The investigator has to make choices about which approach to use and the eventual locations and density of semilandmarks. In studies of growth or evolution of biological material, landmarks represent points that, from prior knowledge, are equivalent in each individual at each stage of developmental or evolutionary transformation. Their differences in relative location over time describe the transformation. However, semilandmarks are located on specimens using algorithms that do not pay regard to development or evolution, and so the consequences of using semilandmarks on resulting analyses of developmental or evolutionary differences in form are unclear. In this study, we compare results among analyses based on landmarks and semilandmarks with each other and with analyses based only on landmarks. We find that while there is some consistency among findings from different semilandmarking approaches, there are also some differences, and that results from such analyses should be considered as approximations of reality that require cautious interpretation.

**Abstract:**

Often, few landmarks can be reliably identified in analyses of form variation and covariation. Thus, ‘semilandmarking’ algorithms have increasingly been applied to surfaces and curves. However, the locations of semilandmarks depend on the investigator’s choice of algorithm and their density. In consequence, to the extent that different semilandmarking approaches and densities result in different locations of semilandmarks, they can be expected to yield different results concerning patterns of variation and co-variation. The extent of such differences due to methodology is, as yet, unclear and often ignored. In this study, the performance of three landmark-driven semilandmarking approaches is assessed, using two different surface mesh datasets (ape crania and human heads) with different degrees of variation and complexity, by comparing the results of morphometric analyses. These approaches produce different semilandmark locations, which, in turn, lead to differences in statistical results, although the non-rigid semilandmarking approaches are consistent. Morphometric analyses using semilandmarks must be interpreted with due caution, recognising that error is inevitable and that results are approximations. Further work is needed to investigate the effects of using different landmark and semilandmark templates and to understand the limitations and advantages of different semilandmarking approaches.

## 1. Introduction

Geometric morphometric (GM) methods are regularly applied in the analysis of size and shape variation among landmark configurations taken from biological structures [1,2,3,4,5]. Landmarks are matched points among objects that define a map of point equivalences among samples. They cannot be readily identified over smooth surfaces such as the cranial vault. It has, therefore, become increasingly common to use algorithms to place densely matched points over such regions in every specimen. In these cases, equivalence of placement is determined by algorithms that often, but not always, use the locations of identifiable landmarks as a guide. While the aim may be explicitly to capture ‘overall form’, e.g., by ‘increasing the density of the shape information’ [6], the issue of homology is still relevant. Algorithmic landmarking methods that use ‘known’ point homologies (landmarks) to estimate ‘dense point correspondences’ (a term commonly used in computer science [7]) among surfaces between landmarks are commonly termed semilandmarking methods or approaches, and such dense point correspondences are known as semilandmarks [8]. This terminology distinguishes them from landmarks that are considered to be equivalent among specimens in the sense of developmental or evolutionary homology [9].

In morphometrics, as applied in biology, methods for semilandmarking have been developed that use equivalent landmarks (based on prior knowledge) as control points to estimate the locations of semilandmarks by projection followed by sliding. Under this procedure, a template specimen is manually landmarked and then semilandmarks are manually or semiautomatically placed on curves and surfaces. Subsequently, the semilandmarks on the template specimen are transferred to each specimen (e.g., by selecting the nearest point between the template and target specimen [10]). This is followed by sliding of semilandmarks, usually to minimise either the bending energy of a triplet of thin-plate splines (TPS) or Procrustes distances among specimens [11,12]. Sliding is achieved iteratively, replacing and refitting the template with the mean for the first iteration, and with the recomputed mean for subsequent ones. Of the two approaches, sliding TPS through the minimisation of bending energy is most commonly applied [13]. It is argued that in Procrustes distance minimisation, all landmarks and semilandmarks influence the sliding, even if very distant from the semilandmarks being slid, while minimisation of bending energy gives greater weight to landmarks and semilandmarks that are local to the semilandmarks that are being slid (note, however, that landmarks may be close to semilandmarks, but on different surfaces). However, in both cases, a set of landmarks is necessary to guide the sliding approach, and it cannot be applied when none are present.

Different strategies for marking up dense point correspondences (=semilandmarks) have been pursued in the field of computer vision. These have been applied to biological material as well as to non-biological objects where they rely on mathematical mappings based on topographic features, rather than developmental or evolutionary equivalences. While the use of topographic features to identify point correspondences is different in principle to how landmarks are said to be identified in biology, in practice, biologists often rely on anatomical features defined topographically rather than through detailed developmental or evolutionary analysis. This is for the simple reason that in closely related species and within species, similar structures in similar locations are usually developmentally and evolutionarily homologous. However, in regions with simple topography, the locations of semilandmarks depend more on the algorithm used to place them. Algorithms used in computer science and biology for mapping include optic flow [7], TPS, and robust point matching (RPM) [14], Generalised Procrustes analysis (GPA) and TPS [15], GPA and coherent point drift (CPD) [16], non-rigid Iterative closest point (NICP) [17,18,19], scaled rigid Iterative closest point (ICP) and visco-elastic models [20], and a 3D registration method integrating ICP, CPD, and the Laplace–Beltrami operator [21]. Notably, in each of these, a set of landmarks (determined algorithmically or visually) provides an initial map of equivalences among specimens that is used to guide subsequent algorithmic marking up of dense correspondences (=semilandmarks) among the surfaces between landmarks.

To avoid manually placing landmarks, several landmark-free algorithms have also been proposed for marking up dense correspondences (=semilandmarks) [22]. The fitting of a template (reference) specimen surface to each target via registration or alignment algorithms underlies the most common approaches, such as the ICP algorithm [23]. This comprises two steps, iterated until the sum of squared distances among point correspondences between the template and target specimens reaches a minimum. First, correspondences are updated by searching for the nearest points to the registered template points in the target. Second, point clouds of template and target surfaces are rigidly registered by minimising the sum of squared Euclidean distances among candidate pairwise corresponding points. Registration and identification of correspondences are then iterated until a minimum is reached. Many variants of the ICP algorithm have been proposed with the aim of improving the accuracy of registration (minimisation of template-target distances) by, e.g., using different distance metrics or assigning different weights to vertices and rejecting outliers [24]. For example, an improved ICP-based approach has been proposed to register the surfaces of specimens by minimising the symmetric point-to-plane distances (along the surface normal vector) instead of point-to-point distances [25]. Additionally, the point correspondences found by the ICP algorithm rely on the initial alignment of two surfaces, by, for example, principal component analysis (PCA), which finds the principal axes of the template and target point sets to provide a sensible initial position. Different approaches lead to different maps of point correspondences (=semilandmarks).

Another landmark-free algorithm, available as an auto3dgm package [26] based on the ICP framework, has been proposed to yield semilandmarks among specimens. In this, a set of points on the template specimen is projected onto the target. However, the choice of the template, the degree of complexity of surfaces, and the density and locations of sampling points affect the results [27,28]. To mitigate this, a template is chosen that has the greatest overall geometric similarity to the members of the sample. Then, semilandmarks of the template specimen are projected to each specimen. Vitek et al. [27] indicated that the choice of initial alignment influences the resulting estimates of point correspondences when using auto3dgm, and the lack of true landmarks as control points impacts registration. Moreover, this approach involves rigid registration and so can result in equivalent points on the template and target specimens being placed on different anatomical features. This is most pronounced among specimens with large differences in shape and size, e.g., points around the zygomatic process of a rigidly registered temporal bone might be projected from the reference onto the condyle of the target [27].

One landmark-free approach [29,30] uses conformal geometry to establish point equivalences among 3D meshes, because any genus zero surface can be mapped conformably onto a sphere, and any surface with a single boundary can be mapped onto a unit disk. In practice, the conformal transformation is applied to the 3D surface and then the correspondences between two surfaces are found in the 2D domain. Examples are provided by the work of [31,32]; however, these conformal methods are sensitive to the quality of surfaces and the complexity of topologies.

Landmark-free algorithms, e.g., the ICP-based method or that described by [33], for marking up point correspondences between surfaces can result in mappings that are quite different from the map of point equivalences based on postulated homologies. This effect can be large, with semilandmarks from the template projected to different anatomical features in the target. In any case, even when ‘appropriately located’, such equivalences have no implicit biological basis; they might, or might not, be good approximations of homology. In consequence, statistical (e.g., PCA) and/or visual (e.g., warping between surfaces) descriptions of (developmental or evolutionary) transformation might or might not properly describe them, and analyses of variation based on them may or may not reflect the developmental or evolutionary basis of variation. By their nature, point correspondences identified without paying attention to homology have an uncertain relationship with the underlying processes responsible for differences in form. They may, however, be useful in discrimination, identification, or classification of specimens to prior groups. These are a different, yet important and common, application of landmark data in computer vision but very different tasks to describing developmental and evolutionary transformations.

It is worth noting that all semilandmarking approaches are dependent on mathematical models of matching. As such, homology is only respected to the extent that the mathematical model uses the homologies of true landmarks to estimate semilandmarks and to the extent that the landmarks actually represent biologically homologous points. Semilandmarks can be intended as estimates of, rather than true, homologous point matchings (landmarks). Thus, different semilandmarking approaches will yield different semilandmarks. The reasons for preferring one approach over another cannot rest entirely on arguments of developmental or evolutionary equivalence of the resulting semilandmarks because the definition and identification of homologous points rely on prior developmental and evolutionary knowledge (which is often lacking). No algorithm without a knowledge-driven model of homology can properly determine or interpolate homology from surface or texture features. Instead, assessment of algorithms has focused on their ‘performance’, defined in various ways.

A few previous studies have attempted to assess the performance of different semilandmarking approaches. Evaluated criteria for comparing different semilandmarking approaches include: the Euclidean distances between semilandmarks (or landmarks) from each approach or with manually placed ones [34]; comparison between methods of the resulting distributions of groups [10,26,35]; the geometric deviation between template and transformed meshes [10,19]; the first two principal components (PCs) [26,35,36]; distance matrices to quantify shape variations [25,26,31,36]; and estimates of centroid size of resulting configurations [34]. These criteria may indicate how different semilandmarking strategies perform in matching surfaces, distinguishing groups, or identifying unknown specimens, but they do not relate to how well the homology map is represented by the resulting semilandmarks. All suffer from the fact that semilandmarks are not point homologies; they may be estimates of such homologies, but each estimate is different. As such, the extent to which they correctly describe biologically homologous anatomical differences and transformations is limited by the extent to which knowledge of homology is embedded in their construction.

The consequences of choosing alternative semilandmarking approaches in studies of biological form variation need to be further investigated. In this study, the degree to which they generate different results is investigated and the significance of the findings for future studies is discussed. To these ends, we employed three landmark-driven semilandmarking methods, sliding TPS, an example of a rigid, and an example of a non-rigid registration approach to yield semilandmarks of varying density. These semilandmarking approaches are compared by assessing the resulting differences in locations of semilandmarks, estimates of mean sizes and shapes, patterns of variation, and covariation in shape with size (allometry). To structure this work, we empirically test six hypotheses using surface scans based on the same template of landmarks and semilandmarks. These are: (i) that for the same density of semilandmarking, there are no differences in semilandmark locations generated by different approaches and (ii) in mean landmark and semilandmark configurations. Furthermore, between densities of semilandmarking using any one approach and between semilandmarking approaches, there are no differences in resulting estimates of (iii) centroid sizes, (iv) distance matrices, (v) PCs of shape variation, and (vi) allometrically scaled shapes. In this study, we do not consider error due to data acquisition or preparation (e.g., imaging modality, segmentation, 3D reconstruction algorithm). Rather, we employ the same 3D surfaces throughout, in order to focus entirely on the differences among semilandmarking approaches applied to them.

## 2. Materials and Methods

### 2.1. Samples

Two high-resolution datasets were used in this study, but only one is reported in the main body of the paper for reasons of brevity. These comprise surface meshes of ape crania and human heads with different degrees of surface complexity and variation in form, and so they allow for an assessment of the extent to which these factors impact on the differences between results obtained using different semilandmarking approaches. The results from the human head are presented in Supplementary Materials, while the focus of the paper is on the results from the ape crania.

#### Ape Crania

**Figure 1:**
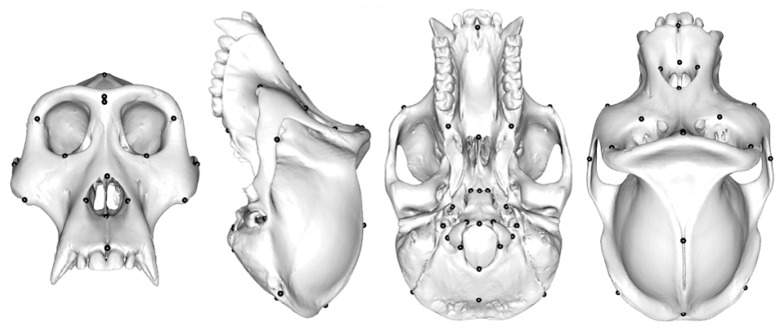
Ape cranial dataset: an ape cranium with 41 anatomical landmarks.

This sample included 20 surface meshes of adult ape crania of both sexes captured by CT scanning, including 5 *Gorilla*, 5 *Hylobates lar*, 5 *Pan troglodytes*, and 5 *Pongo abelii*. Each ape cranial surface model comprises more than 196,000 vertices and 391,000 triangle meshes. These present more complex surfaces and a far greater degree of variation in size and shape than the head surface dataset (see Supplementary Materials). As shown in Figure 1, 41 anatomical landmarks, presumed to be homologous, had already been manually placed over the entire cranium in the course of a previous study [37].

### 2.2. Methods

#### 2.2.1. Three Semilandmarking Approaches

Based on the fixed landmarks, we used three different methods to mark up semilandmark sets of varying density among specimens using a common template. These are sliding semilandmarks using thin-plate splines, a hybrid method comprising thin-plate splines and the NICP algorithm, and an approach using least squares (LS) and the ICP algorithm.

##### Generation of the Template

A landmark and semilandmark template was created to be used as the basis of semilandmarking the ape crania using three different approaches. The external surface of every specimen was extracted to avoid the internal surface interfering with the sliding and projection of semilandmarks. Next, we used the k-means clustering algorithm to evenly sample 800 surface semilandmarks over an arbitrary specimen (a male gorilla), ignoring symmetry. The k-means clustering algorithm was applied to the vertex coordinates of the template surface to obtain k sub-regions. Next, the centroids of each sub-region were projected onto the template surface by searching the nearest point. Ultimately, these projected points over the template were treated as surface semilandmarks. Using the template, we employed the sliding TPS approach to yield semilandmarks among specimens. Then, the mean form of landmarks and semilandmarks was calculated, and the arbitrary specimen surface was deformed to approximate the mean surface form. This surface was used as the template. Finally, we utilized the k-means clustering algorithm to evenly sample five different densities of surface semilandmarks (50, 100, 200, 400, 800) on the template, avoiding the cranial base and teeth. Note that the Procrustes distance between the arbitrary specimen and the template based on the mean landmarks and 800 semilandmarks was 0.0988, which is very similar to the average difference between individuals and the mean, estimated using sliding TPS semilandmarking.

##### Semilandmarking Approaches

Once the template was created, three methods of semilandmarking were applied as follows:

(a) Sliding TPS

Semilandmarks were projected from the template surface onto each target surface and then iteratively slid over the target surface to minimise the bending energy of the TPS between each specimen and template. This is the classic approach, first described by [8] and developed by [11,13]. We used the patching (placePatch) and sliding (slider3d) procedures in the Morpho R package to slide the semilandmarks on the templates [38].

(b) Rigid registration

Rigid registration aims to find the linear transformation of the template to each specimen surface (translation and rotation) that aligns two surfaces without scaling, in such a way that the sum of squared Euclidean distance between landmarks (and, if present, semilandmarks) is minimised. Note that this method does not deform a surface to optimise fitting, so it is prone to error due to the difference in size between the template and every specimen.

Semilandmarking of the target was achieved iteratively using a hybrid rigid registration combining LS and point-to-point ICP algorithms (LS&ICP). First, the LS algorithm was used to fit the template to each specimen, minimising the distances between landmarks on the template and each target specimen by translating and rotating the template to best fit the target. Subsequently, the ICP algorithm iteratively rigidly refitted the template to the target by minimising the sum of the squared distances between the landmarks and current estimate of semilandmarks, found by searching for the nearest points on the target surface from the registered template semilandmarks. The initial rigid alignment based on landmarks speeds up convergence during the ICP phase.

(c) Non-rigid registration

We used a hybrid non-rigid registration approach [39] to deform the template specimen to fit each target specimen and then projected the semilandmarks from the warped template onto each specimen by searching for the nearest points on its surface, to yield semilandmarks across all specimens. Unlike rigid registration, in fitting, each vertex of the template can be moved freely with stretching based on a non-rigid transformation and landmarks acting as constraints. This comprised two steps: First, the TPS algorithm was used to warp the template to every specimen based on the fixed landmarks. This removed size and shape differences between the template and each target set of landmarks and provided a reasonable initial alignment of surfaces. Second, the NICP algorithm [17] was applied to warp the deformed template surface to each specimen as rigidly as possible, optimizing the cost function to assign an affine transformation to each vertex, rather than an interpolation function as used in TPS. For this procedure, the cost function comprised a landmark term, a local affine regularization term, and a stiffness term. Registration loops were performed by decreasing stiffness weights iteratively and deforming the template incrementally. This resulted in the warped template surface matching the target closely. Here, this approach is referred to as TPS&NICP.

#### 2.2.2. Comparison of Three Semilandmarking Approaches

We compared the different semilandmarking approaches by testing the null hypotheses described in the Introduction. These relate to differences in locations of semilandmarks, estimates of mean sizes and shapes, patterns of variation, and covariation in shape with size (allometry).

##### The Locations of Semilandmarks

Differences between methods in the placement of semilandmarks were assessed by visualizing them and computing the Euclidean distances between each semilandmark (that share the same initial template position), computed using each semilandmarking approach. These were used to compute the average semilandmarking ‘error’ between approaches and to examine their distributions. Note that these are ‘errors’ between algorithmic results and not in homology mapping per se, which cannot be evaluated because truly homologous dense point correspondences are unknowable.

##### Comparisons of Mean Landmark and Semilandmark Configurations

Generalised Procrustes analysis was applied to the landmark and semilandmark configurations estimated for the sample, and then the mean centroid sizes were compared among different semilandmarking methods and densities. Subsequently, the Procrustes distances among estimates of the mean shape were computed to quantify the differences between them arising from different semilandmarking approaches. A permutation test with 1000 runs was performed to assess the significance of differences between the mean shapes generated by different approaches. To contextualise the extent to which Procrustes distances between means differ, these were compared to the average distances between individuals and the mean for each density of semilandmarks.

##### Procrustes Distances among Specimens Obtained Using Different Semilandmarking Approaches and Densities

We examined the effect of different semilandmarking approaches and different densities of semilandmarks on Procrustes distance matrices.

(a) The effect of different semilandmarking approaches

Generalised Procrustes analysis was applied to each of the landmark and semilandmark sets generated by the different semilandmarking approaches. Then, Procrustes shape coordinates and their sample means and centroid sizes were obtained. Procrustes distance matrices among all individuals were calculated and a Mantel Test [40] performed to compare distance matrices obtained by the different semilandmarking approaches. Additionally, vectors of Procrustes distances between each individual and the mean were compared among semilandmarking methods by plotting bivariate graphs and computing Pearson correlations among them.

(b) The effect of different densities of semilandmarks

The results obtained from analyses of the landmarks and different densities of semilandmarks were compared with those obtained using the landmarks and maximum density of semilandmarks from each method. This was achieved by computing the Pearson correlations among vectors of Procrustes distances to the mean and by performing a Mantel Test between the Procrustes distance matrices derived from each density and that from the maximum density.

Additionally, the matrix of Procrustes distances among specimens based on the landmarks alone was computed in order to compare these distances with those obtained by different semilandmarking approaches and densities.

##### PCA and Allometry

For the landmarks alone, a GPA followed by a PCA of the covariance matrix were carried out in order to compare PCs with those from the semilandmarking methods. Then, for the landmarks and each density of semilandmarking, a separate GPA was carried out of the landmark and semilandmark configurations derived from each of the three semilandmarking methods. A PCA was then carried out on the resulting shape coordinates from each GPA at each semilandmarking density. To assess how the major vectors of variation (PCs) differ between approaches and semilandmarking densities, we compared the distributions of specimens along the first two PCs by computing the Pearson correlations and parametric tests of the significance of these correlations among the PC1 and PC2 scores arising from each semilandmarking approach and from landmarks alone.

Next, a joint GPA and PCA was carried out combining landmarks and semilandmark sets of the same density from each semilandmarking approach. Allometry [41] was estimated for the whole sample and each semilandmarking density by multivariate regression of the full set of PC scores on the natural logarithm of centroid size. These estimates of allometry were then compared between different semilandmarking approaches based on the angles between allometric vectors [42]. Small angles indicate that semilandmarks generated by different approaches are similar, and large angles indicate that they are more different. A permutation test with 1000 runs was performed to assess the significance of the angles between allometric vectors derived from different semilandmarking approaches.

Subsequently, the predicted shapes of landmarks and semilandmarks representing the extreme limits (smallest-largest) of the allometric vectors derived using each semilandmarking method and density were compared. This comprised two steps: First, the predicted shapes corresponding to the upper and lower limits of centroid sizes estimated by each approach were estimated from the multivariate regression [1]. Second, Procrustes distances were computed between the predicted shapes representing the maximum and minimum centroid sizes from each of the landmark and semilandmark sets generated by the different semilandmarking approaches.

## 3. Results

### 3.1. The Locations of Semilandmarks

**Figure 2:**
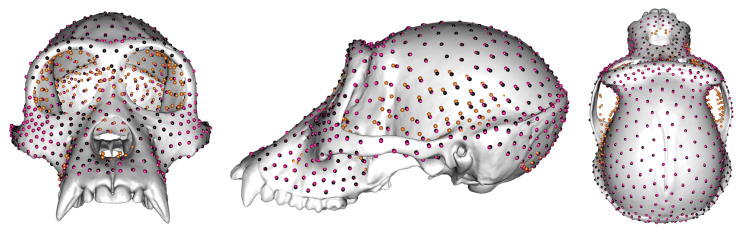
800 semilandmarks generated by sliding TPS (black points), LS&ICP (amber points), and TPS&NICP (magenta points) approaches on the mean surface generated by sliding TPS.

Figure 2 shows the average locations of 800 semilandmarks generated by sliding TPS (black points), LS&ICP (amber points), and TPS&NICP (magenta points) approaches on the mean surface generated by deforming the template cranium to the mean landmarks and semilandmarks derived from the sliding TPS approach. Semilandmarks generated by LS&ICP tend be located in different positions to those from the other approaches. Additionally, the mean configurations from the TPS&NICP and, particularly, LS&ICP approaches do not exactly lie on the sliding TPS mean surface. These differences are particularly evident for semilandmarks around and in the orbits, temporal fossae, over the brow ridges, zygomatic arch, and maxilla.

**Figure 3:**
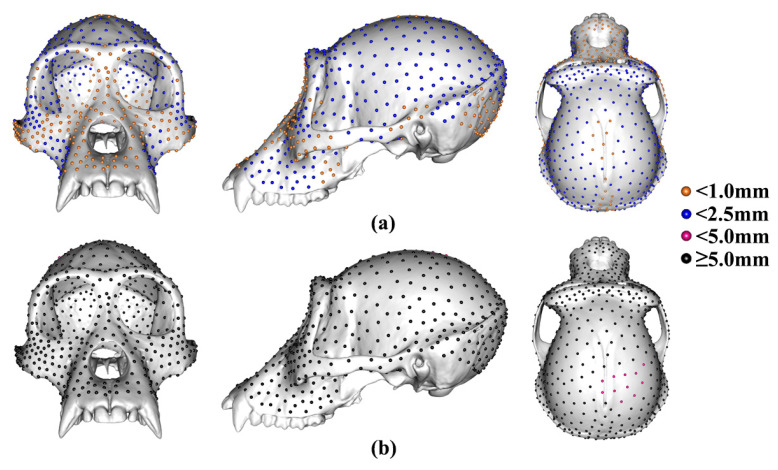
The average differences in location (mm) between 800 semilandmarks generated by different approaches. (**a**) Differences between sliding TPS and TPS&NICP approaches. (**b**) Differences between sliding TPS and LS&ICP approaches. Differences between TPS&NICP and LS&ICP approaches are not shown because they are very similar to those in (**b**).

Table 1 shows the differences in locations of semilandmarks generated by sliding TPS and TPS&NICP approaches. It lists average differences in location (dev in mm) and the percentage of semilandmarks (%) that differ in location by 0.0–1.0 mm (amber points), 1–2.5 mm (blue points), 2.5–5.0 mm (magenta points), and ≥5.0 mm (black points). Figure 3 illustrates these differences. Between sliding TPS and TPS&NICP approaches, differences are all less than 5.0 mm, with the majority (>99%) less than 2.5 mm (Figure 3a), and the proportion of semilandmarks from sliding TPS and TPS&NICP located within 1.0 mm of each other tends to decrease with increasing semilandmarking density. By contrast, the semilandmark locations derived from LS&ICP are more different from those derived by both sliding TPS and TPS&NICP. For brevity, only the differences in semilandmarks between sliding TPS and LS&ICP (Table 2 and Figure 3b; 98.62% ≥5.0 mm) are presented, but the results are similar for the comparison of TPS&NICP with LS&ICP.

### 3.2. Differences among Mean Landmark and Semilandmark Locations

To compare the estimates of mean landmark and semilandmark configurations, Procrustes distances were computed among their resulting mean landmark and semilandmark configurations (Table 3). The Procrustes distances increase with increasing density. The estimates of the mean landmark and semilandmark configurations generated from sliding TPS are more similar to TPS&NICP. There is no significant difference in mean landmark and semilandmark configurations between the means derived from sliding TPS and TPS&NICP approaches. Procrustes distances range from 0.0051 with 50 semilandmarks to 0.0072 with 800 semilandmarks, which are 4.87% and 7.62% of the average distance (computed using semilandmarks derived from sliding TPS plus landmarks) of specimens from the mean at each density. The LS&ICP approach produces quite different means to those obtained using the other semilandmarking approaches, especially at higher semilandmarking densities.

### 3.3. Comparison of Centroid Sizes and Procrustes Distance Matrices

The differences in semilandmark locations derived using LS&ICP from those derived by the other two approaches are emphasised by the analyses in Table 4. This table presents the Pearson correlations among the vectors of Procrustes distances between every individual and the mean as well as the correlations, from Mantel Tests, comparing the matrices of Procrustes distances among all individuals, calculated using landmarks and semilandmarks generated by different semilandmarking approaches and densities. The largest (all > 0.99) Pearson and Mantel correlations were found between sliding TPS and TPS&NICP. LS&ICP clearly produces quite different results to those obtained using the other semilandmarking approaches, especially at higher semilandmarking densities.

Pearson correlations were computed among vectors of Procrustes distances between each individual and the mean, as were Mantel correlations among the matrices of Procrustes distances. These, presented in Table 5, compared the distances from the landmark and 800 semilandmark configuration with those from configurations comprising 50–400 semilandmarks. Within each semilandmarking approach, these correlations are generally large (>0.90) and increase with increasing numbers of semilandmarks. The largest correlations are found across semilandmarking densities arising from TPS&NICP and the smallest from LS&ICP. Additionally, Table 6 presents Pearson correlations between the distance vectors and matrices calculated using the full set of landmarks alone, and those from the landmarks and semilandmarks generated by each semilandmarking approach and density. Distances from sliding TPS and TPS&NICP approaches are similarly correlated with those from landmarks alone. Thus, these correlations are ~0.96 for the lowest density of semilandmarking and fall gradually to ~0.9 for the highest. In contrast, correlations for the LS&ICP approach are moderate (~0.5–0.75) but follow the same trend by becoming smaller with increasing semilandmarking density.

**Figure 4:**
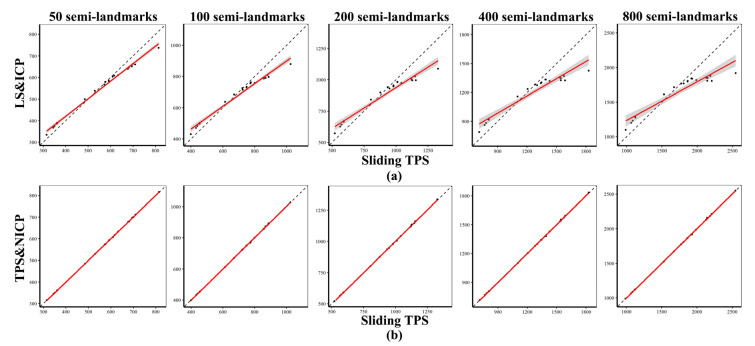
Comparisons of centroid sizes estimated by different semilandmarking approaches. (**a**) Sliding TPS vs. LS&ICP. (**b**) Sliding TPS vs. TPS&NICP.

**Figure 5:**
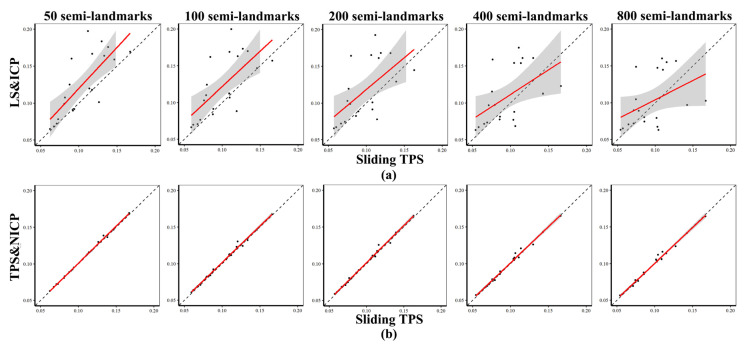
Comparison of the vector of Procrustes distances from the mean between semilandmarking approaches. (**a**) Sliding TPS vs. LS&ICP. (**b**) Sliding TPS vs. TPS&NICP.

**Figure 6:**
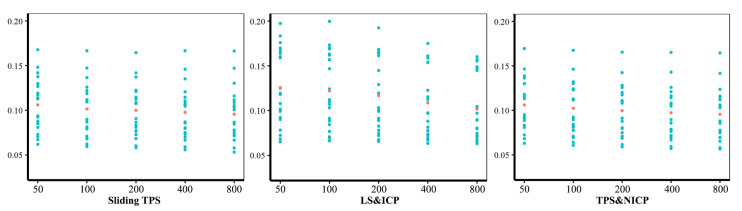
Vectors of Procrustes distances between each individual and the mean computed for each semilandmarking approach using different densities of semilandmarks. Cyan points represent Procrustes distances between every specimen and the Procrustes mean shape and red points represent the average value of the Procrustes distance vector.

Centroid sizes and Procrustes distances between each cranium and the mean were very similar between landmark and semilandmark configurations from sliding TPS (horizontal axis) and TPS&NICP (vertical axis), as indicated in Figure 4 and Figure 5, where the fitted lines are almost coincident with the dashed lines, thereby indicating identity. However, LS&ICP tends to produce landmarks and semilandmark configurations with larger centroid sizes than those from sliding TPS for small crania, and smaller for large crania (Figure 4). Comparisons of Procrustes distances from the mean derived from LS&ICP and sliding TPS (Figure 5) at varying semilandmark densities show marked differences, unlike comparisons between the same distances from TPS&NICP and sliding TPS. Figure 6 summarizes the vectors of Procrustes distances between each individual and the mean calculated using landmarks and semilandmarks from the sliding TPS, LS&ICP, and TPS&NICP approaches. The sliding TPS and TPS&NICP methods are consistent across semilandmarking densities, whilst the LS&ICP approach results in different estimates of centroid size and Procrustes distances.

### 3.4. PCA and Allometry

**Figure 7:**
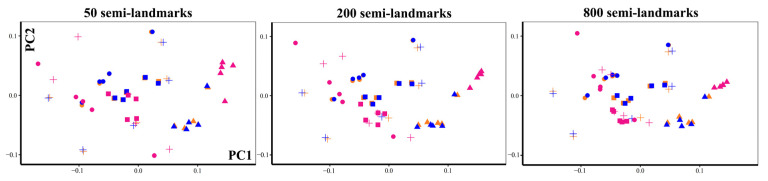
Visualization of superimposed scatterplots of PC1 and PC2 from analyses of 20 ape crania using landmarks and semilandmarks from sliding TPS, LS&ICP, and TPS&NICP approaches with varying densities. The horizontal axis represents PC1 and the vertical, PC2. Cross: *Pongo abeli*. Circle: *Gorilla*. Rectangle: *Pan troglodytes*; Triangle. *Hylobates lar*. Amber: Sliding TPS. Blue: TPS&NICP. Magenta: LS&ICP.

We calculated the correlations (ignoring sign, and so arbitrary reflections of PCs) of scores on the first two PCs of shape variation resulting from the separate GPA and PCA of each semilandmark configuration and density (Table 7). The correlations among PC1 and PC2 scores from sliding TPS and TPS&NICP are all greater than 0.99. The scatterplots of the first two PCs reflect this and account for ~60% of the total shape variance. In contrast, the correlations among PC1 and PC2 scores are lower between LS&ICP and the other two methods and become smaller with increasing density, especially for PC2 scores. Plots of the first two PCs form separate analyses of the landmarks and semilandmarks arising from each approach are superimposed in Figure 7. Scatterplots of PC1 and PC2 generated by sliding TPS (amber) coincide with those obtained by TPS&NICP (blue), while LS&ICP (magenta) produces quite different PC1 and PC2 scores to those obtained using the other semilandmarking approaches.

Table 8 presents the correlations between PC scores on the first PCs of shape from each landmark and semilandmark configuration generated by the different semilandmarking approaches and densities and those from GPA and PCA of the landmarks alone. These are very similar for the sliding TPS and TPS&NICP semilandmarking approaches, being large to moderate and slightly greater for PC1 than PC2 scores, decreasing ~5–7.5% with increasing semilandmarking density. The correlations of PC1 scores from LS&ICP are stable with increasing density, while the correlations of PC2 scores dramatically decline. Additionally, the correlations of PC1 and PC2 scores between each semilandmarking density and the maximum (800) density are presented in Table 9. This shows that the correlations are high and increase with increasing density.

The angles between allometric vectors derived by multivariate regression of shape (the full set of non-zero PC scores) on the natural logarithm of centroid size using landmarks and semilandmarks from sliding TPS and TPS&NICP (Table 10) are generally small (<9°) and increase moderately with semilandmarking density. There is no significant difference in angles between allometric vectors generated by sliding TPS and TPS&NICP approaches. By contrast, the angles between allometric vectors generated by LS&ICP and the other two methods are greater and increase with increasing semilandmark density.

Because the LS&ICP approach results in allometric vectors that differ significantly from those arising from the other two semilandmarking approaches, the comparison of allometrically scaled shapes among ape crania focuses on the differences between the sliding TPS and TPS&NICP approaches. The Procrustes distances between predicted landmark and semilandmark configurations at the extreme limits of the allometric vector are shown in Table 11. The Procrustes distances between allometric predictions of cranial shape at the maximum centroid size are between a half and two-thirds of those between predictions at the minimum centroid size. This is explained by the distribution of centroid sizes being skewed towards the maximum (as with the head surface data; see Supplementary Materials). These distances increase with semilandmarking density. They range between 9% and 14% of the average distance of specimens from the mean for the predictions of cranial shape at the maximum centroid size and between 16% and 21% for predictions at the minimum centroid size.

## 4. Discussion

This study compares alternative strategies for marking up dense point correspondences (=semilandmarks) among biological structures for subsequent statistical analyses. We compared these semilandmarking approaches by empirically testing six null hypotheses that there are no differences between semilandmarking approaches in semilandmark locations, estimates of mean form, patterns of variation, and co-variation of shape with size (allometry). These hypotheses are all falsified, as expected, but the results of each analysis provide quantitative insights into the nature and degree of difference in results obtained by each semilandmarking approach.

### 4.1. Significance and Implications of Findings

Thus, in the present and previous studies, differences are found in the locations of semilandmarks produced using different approaches and these have consequences for subsequent analyses. Mean landmark coordinates, centroid sizes, and distributions of specimens in Kendall’s shape space are all impacted by the locations of semilandmarks. Further, just as different landmarking choices impact the results of subsequent analyses, so do variations in the number and locations of semilandmarks.

Previous studies have noted that with increasing semilandmark density, there is increasing consistency of scores on PC1 [26,27], while [35] found that increasing density of semilandmarks did not necessarily result in greater group separation. These studies did not assess consistency of results with increasing density, among alternative semilandmarking methods. Here, we find that the results generated by sliding TPS and TPS&NICP approaches are most consistent. Greater differences are found between landmarks and semilandmarks from LS&ICP and the other two approaches, especially for the more complex surfaces of the ape crania (compared with the head surface data, see Supplementary Materials). For the ape cranial data, sliding TPS and TPS&NICP produce distributions that are consistent within semilandmarking approaches across densities. As such, TPS&NICP produces the most consistent results with both surface datasets and between semilandmarking densities. Sliding TPS is almost as consistent when applied to surfaces with landmarks over their entirety.

However, consistency does not relate to how well the homology map is represented by the resulting semilandmarks. Methods may be consistently wrong in identifying homology, and so in describing differences. Here, for instance, both sliding TPS and TPS&NICP use a triplet of thin-plate splines to achieve an initial fit between the template and each specimen. In consequence, these algorithms begin with initial placements of semilandmarks that are identical. This could well underlie why these two approaches achieve very similar results, rather than because they both converge on ‘the correct solution’. Each method estimates equivalent points in terms of its specific algorithm, but each estimate is different. In fact, all estimates of mean coordinate configurations, of the distance matrices and other statistical results, are correct in each analysis, insofar as they are the correct results obtained from the landmarks and semilandmarks. Differences arise because of differences in the data in the landmark and semilandmark locations. The issue in studies that aim to describe and compare developmental or evolutionary transformations is which, if any, of the semilandmarking approaches correctly mark up homologies. How well the resulting semilandmarks represent homologies among specimens is limited by the extent to which knowledge of homology is applied in locating them, by the fact that such point homology is largely unknowable and, indeed, may not exist in reality because points at one stage may not actually turn into points at another. These considerations also apply to landmarks themselves, albeit arguably to a lesser degree.

Semilandmarks have deficient coordinates [11] and so are located on the surface of interest but with uncertainty regarding the equivalence (e.g., homology) of their position. The authors of [5,9,43] argued that the locations of the semilandmarks themselves should not be interpreted but rather the form of the surface mesh or curve that they describe should be the basis of comparison. This recognises their deficient coordinates in focusing on the form of the surface itself; however, it also raises an important point and a question.

Thus, semilandmarks describe surfaces, but different semilandmarking approaches achieve this through different locations of semilandmarks. These differences in location have effects on subsequent statistical analyses, here resulting in estimates of mean configurations, distributions and principal modes of shape variation (PCs), and covariation (e.g., allometry) that differ to some degree (also see analyses of head surfaces in Supplementary Materials). This is an important point, because we rely on statistical results to test our hypotheses, and yet, where these concern developmental or evolutionary transformations, the extent to which analyses of shape variation and covariation using any one method or density of semilandmarking respect and reflect homology is also unknowable.

Some insight into this might be gleaned from a consideration of how well findings from analyses of landmarks and semilandmarks match findings based on presumed homologous points alone. Here, several analyses have compared results obtained by different semilandmarking approaches and densities with those from landmarks alone. For ape crania, where landmarks are located, albeit sparsely, over the whole surface, Procrustes distances from both sliding TPS and TPS&NICP correlate strongly (>0.9) with distances from landmarks (Table 6). For the head surface data (see Supplementary Materials), correlations are weaker, but for the face alone, where landmarks are present, correlations are moderate to high. These findings suggest that analyses of landmarks and semilandmarks are consistent with those of landmarks alone, when landmarks are sufficient in number and located such that they delimit the surfaces that are to be semilandmarked. There are some consistent differences, which, as noted above, may be due to better description (additional information) of surface form or to shared error. However, given that high-dimensional data, such as arises with semilandmarks, present serious analytical issues [44], the potential benefits of semilandmarks, particularly in visualisation, should be set against the potential pitfalls of statistical analysis of such data [45]. The statistical gains are, at best, unclear in the analyses presented here, and there is an unresolvable doubt that the ‘gains’ may, in fact, not be gains at all, but rather due to consistent erroneous identification of homologies between approaches. There may, however, be gains in applications to discrimination, identification, and classification [46,47], but these topics are not considered here, and further studies need to be conducted to assess this possibility.

With regard to visualisation, semilandmarks are often applied to enable detailed high-quality representation of results as surface warpings. Surface mesh form, rather than the form of a landmark and semilandmark configuration over the surface, is relevant in many practical circumstances. For example, surfaces are often visualised by warping a template to statistical estimates of, e.g., mean landmark and semilandmark configuration form [48]. Beyond this, surfaces representing statistical results, such as the mean, might be used in the clinic to compare patient cranial form with that of the wider population, using clinic and condition-specific (re)parameterisations of reference and patient surfaces [49]. Another increasingly common application of surfaces arising from geometric morphometric analyses is to use them to build finite element models [50,51]. Thus, an important question arises, which is considered in a follow-on study [52]: how do different semilandmarking approaches perform in characterising the form, variation, and covariations of the shape of the surface mesh itself, rather than the locations of semilandmarks on it?

What are the implications of this study for future work using semilandmarks? The results indicate varying degrees of consistency in results derived by different semilandmarking approaches and densities. However, as has been noted above, consistency does not necessarily indicate reliable identification of homologous points. This echoes [45], who noted that consistency might be thought of as suggesting precision (repeatability of measures) but does not equate with accuracy (i.e., correctly marking up homologous points).

It is not possible to state that any one method is superior to any other in identifying homologous semilandmarks, but it is clear from our findings with LS&ICP that some methods result in semilandmarks that clearly do not represent homologies, while estimates from sliding TPS and TPS &NICP appear more reasonable in anatomical terms. Thus, in applying any method, extrinsic anatomical knowledge can guide assessment of accuracy of semilandmarking *sensu* [45], but this is subjective. Some approaches will clearly fail this test while others will not. However, every approach will give rise to different statistical results. The extent to which differences due to the choice of semilandmarking approach are important depends on the how large they are in relation to the aspects of variation among specimens that are of interest.

The degree to which results from semilandmarks are correlated with those from landmarks alone might be used as a basis for identifying ‘good’ methods (that yield results consistent with those based on homologous landmarks), but perfect association between methods would rather undermine the need for analyses of semilandmarks in the first place. This is similar to the situation with true landmarks, in that landmarks can be located with error and different sets of landmarks may be chosen, and both affect statistical results. However, landmarks, unlike semilandmarks, being defined based on prior anatomical knowledge, do not require an algorithm to locate them. As noted by [9], the number and locations of landmarks chosen in any particular study can and should be based on the question at hand. Many questions can be sensibly and fully addressed using a few well-chosen landmarks. However, as noted in the Introduction, landmarks may be sparse on (homologous) structures of interest; they can also have doubtful homology or be difficult to locate.

A cautious approach to working with landmark and semilandmark data would be, first, to design a landmark configuration that relates to the hypothesis under test [9] and then semilandmark the sample. Statistical testing might then be seen as distinct from visualisation and proceed on the basis of the landmark configurations alone. Visualisations (warped surface meshes between, e.g., means or representing a vector of transformation) can then be estimated based on parallel analyses using the landmarks and semilandmarks. This avoids the philosophical issues that arise concerning semilandmark homology, and it avoids the statistical issues that arise when many variables are taken on small samples than arise with semilandmarks (p (number of variables)/n (sample size) ratio) [44,45]. However, this approach limits the analysis to identifiable landmarks and so omits what might be a useful ‘signal’ from the surface between landmarks. In semilandmarking surfaces, there is a decision to be made regarding the balance between the likelihood of erroneous results (‘noise’—inaccurate identification of homologous points and the ratio of the number variables to the number of specimens—p/n ratio) from semilandmarking and the potential gains in ‘signal’. To a large degree, this is a judgement call. However, the p/n ratio issue can be mitigated by minimising the number of semilandmarks used, while the issue of homology of semilandmarks cannot.

### 4.2. Limitations and Future Work

As noted in the Introduction, previous studies have compared the performance of different semilandmarking approaches based on different criteria: distance matrices [25,26,31], principal components (PCs) [10,13,26,35], and differences between template and transformed meshes [10,19]. Such sensitivity studies are useful in understanding sources of error and in guiding eventual parameterisation in a particular context, but it is not clear how generalisable their findings are because each is empirical. This caveat also applies to the present study and so its findings cannot be considered as definitive; rather, they offer insights into the consistency of statistical findings based on a limited range of alternative semilandmarking approaches.

This study is limited in its scope, having examined only three possible semilandmarking approaches applied to head and cranial surface data. Future work should consider a wider range of surface data, alternative ‘landmark free’ approaches (e.g., [33]), the effects of varying numbers of landmarks on semilandmarking, and the consequences of varying semilandmark locations on the template at specific semilandmarking densities. Additionally, because different semilandmarking approaches generate different semilandmark locations and these affect the distributions of data, it is likely that there will be consequences for statistical tests. Thus, studies need to be conducted using real and simulated data, created by perturbing a known surface in known ways to allow for assessment of the precision and accuracy of estimation of means and other statistical parameters. Finally, it would be of interest to more widely explore the consequences of different semilandmarking approaches in comparing allometric trajectories between sexes or species. This was not possible in the present study, which was limited to comparing predicted allometrically scaled mean shapes and the angles between allometric vectors derived using different semilandmarking approaches.

## 5. Conclusions

In summary, this study utilized three different semilandmarking approaches to yield semilandmarks at different densities. The effects of different semilandmarking approaches and densities of semilandmarks on semilandmark locations and on subsequent statistical results were then considered. It is not possible to assess the extent to which the different approaches yield semilandmarks that accurately reflect homology, but it was possible to assess the consistency (=precision) [45] between approaches and densities of semilandmarks. The TPS&NICP approach yields the most consistent results across varying semilandmark densities applied to both the head surface and ape cranial data, and sliding TPS produces results that are most consistent with those from TPS&NICP. However, consistency is not the same as accuracy and so it is not possible to say which, if any, method(s) produce semilandmarks that accurately represent homologies among specimens. This is a significant issue in applications to the study of developmental or evolutionary transformations but less so in other applications, such as identification/discrimination. By focusing on landmarks with more secure homology for statistical analyses and employing semilandmarks for visualisation, these issues are minimised.

Further work is needed to assess alternative semilandmarking approaches in different contexts. While the sliding TPS and TPS&NICP algorithms give rise to very similar findings in this study, further work, as described in the previous section, is needed to fully understand the limitations of each approach and no specific recommendation on choice of method in specific contexts can yet be made. For now, interpretations of statistical results based on semilandmarks should be made with due caution regarding the potential errors in semilandmarking, and serious consideration should be given to why semilandmarking is being undertaken, given that simpler landmark data may well yield the same results, with less uncertainty about homology and so interpretation of studies of transformation of form.

## Figures and Tables

**Table 1:** Comparison of semilandmarks from sliding TPS and TPS&NICP approaches.

diff. mm	50		100		200		400		800	
	dev	%	dev	%	dev	%	dev	%	dev	%
[0.0–1.0)	0.56	52.00	0.64	46.00	0.70	35.50	0.68	30.25	0.70	33.75
[1.0–2.5)	1.34	48.00	1.44	54.00	1.47	64.00	1.49	69.50	1.50	66.13
[2.5–5.0)	-	-	-	-	2.99	0.5	2.57	0.25	2.73	0.12
≥5.00	-	-	-	-	-	-	-	-	-	-
Total	0.94	100.00	1.08	100.00	1.21	100.00	1.25	100.00	1.23	100.00

**Table 2:** Comparison of semilandmarks from sliding TPS and LS&ICP approaches.

diff. mm	50		100		200		400		800	
	dev	%	dev	%	dev	%	dev	%	dev	%
[0.0–1.0)	-	-	-	-	-	-	-	-	-	-
[1.0–2.5)	-	-	-	-	-	-	-	-	-	-
[2.5–5.0)	4.93	2.00	4.54	4.00	4.58	5.50	4.82	1.25	4.80	1.38
≥5.00	9.04	98.00	9.89	96.00	10.41	94.50	11.05	98.75	11.52	98.62
Total	8.96	100.00	9.68	100.00	10.09	100.00	10.97	100.00	11.37	100.00

**Table 3:** Procrustes distance (permutation test *p* < 0.05 *) between mean landmark and semilandmark configurations derived at varying densities.

	50	100	200	400	800
Sliding TPS LS&ICP	0.0419	0.0532	0.0554 *	0.0582 *	0.0581 *
Sliding TPS TPS&NICP	0.0051	0.0061	0.0072	0.0067	0.0072
LS&ICP TPS&NICP	0.0419	0.0534	0.0556 *	0.0589 *	0.0591 *

**Table 4:** Pearson correlations (all *p* < 0.01, except ^#^, *p* < 0.05, * = n.s., parametric test) among the vectors of Procrustes distances between each ape cranium and the mean and Mantel tests among Procrustes distance matrices.

	50		100		200		400		800	
	Pearson	Mantel	Pearson	Mantel	Pearson	Mantel	Pearson	Mantel	Pearson	Mantel
Sliding TPS LS&ICP	0.7630	0.8024	0.6789	0.7403	0.5954	0.6711	0.5066 ^#^	0.5993	0.4185 *	0.5241
Sliding TPS TPS&NICP	0.9988	0.9986	0.9962	0.9970	0.9959	0.9961	0.9951	0.9947	0.9948	0.9944
LS&ICP TPS&NICP	0.7540	0.7929	0.6643	0.7268	0.5806	0.6561	0.4739 ^#^	0.5761	0.3881 *	0.5050

**Table 5:** Pearson correlations (all *p* < 0.001, parametric test) among vectors of Procrustes distances between each individual and the mean and Mantel tests comparing Procrustes distance matrices between each density of semilandmarking and the maximum density.

	50		100		200		400	
	Pearson	Mantel	Pearson	Mantel	Pearson	Mantel	Pearson	Mantel
Sliding TPS	0.9770	0.9752	0.9907	0.9899	0.9945	0.9945	0.9991	0.9990
LS&ICP	0.8931	0.9123	0.9324	0.9453	0.9688	0.9752	0.9918	0.9931
TPS&NICP	0.9849	0.9833	0.9943	0.9937	0.9973	0.9974	0.9993	0.9994

**Table 6:** Pearson and Mantel correlations (all *p* < 0.01, except ^#^, *p* < 0.05) between vectors and matrices of Procrustes distances from each semilandmarking approach and density and those from the landmarks alone.

	50		100		200		400		800	
	Pearson	Mantel	Pearson	Mantel	Pearson	Mantel	Pearson	Mantel	Pearson	Mantel
Sliding TPS	0.9619	0.9579	0.9460	0.9401	0.9303	0.9244	0.9160	0.9079	0.9105	0.8995
LS&ICP	0.7424	0.7951	0.6627	0.7532	0.6081	0.7153	0.5413 ^#^	0.6742	0.4916 ^#^	0.6402
TPS&NICP	0.9602	0.9551	0.9473	0.9391	0.9350	0.9241	0.9260	0.9135	0.9221	0.9076

**Table 7:** Comparison of Pearson correlations (all *p* < 0.01, except ^#^, *p* < 0.05, * = n.s.) of PC1 and PC2 between sliding TPS and TPS&NICP.

	50		100		200		400		800	
	PC1	PC2	PC1	PC2	PC1	PC2	PC1	PC2	PC1	PC2
Sliding TPS LS&ICP	0.9400	0.9387	0.8981	0.8119	0.8561	0.6647	0.8163	0.4961 ^#^	0.7666	0.3334 *
Sliding TPS TPS&NICP	0.9993	0.9989	0.9983	0.9992	0.9978	0.9985	0.9974	0.9977	0.9972	0.9967
LS&ICP TPS&NICP	0.9357	0.9264	0.8919	0.8030	0.8443	0.6610	0.7988	0.4749 ^#^	0.7498	0.3291 *

**Table 8:** Pearson correlations (all *p* < 0.01, except ^#^, *p* < 0.05, * = n.s.) among PC scores from each semilandmarking density and from the landmarks alone.

	50		100		200		400		800	
	PC1	PC2	PC1	PC2	PC1	PC2	PC1	PC2	PC1	PC2
Sliding TPS	0.9487	0.8634	0.9238	0.8435	0.9104	0.8188	0.9021	0.8155	0.8963	0.8077
LS&ICP	0.9399	0.7606	0.9329	0.6427	0.9291	0.5515 ^#^	0.9228	0.4363 *	0.9116	0.3325 *
TPS&NICP	0.9430	0.8732	0.9186	0.8434	0.8999	0.8020	0.8880	0.8108	0.8833	0.8012

**Table 9:** Pearson correlations (all *p* < 0.01, parametric test) among PC scores from each semilandmarking density and the maximum density.

	50		100		200		400	
	PC1	PC2	PC1	PC2	PC1	PC2	PC1	PC2
Sliding TPS	0.9888	0.9769	0.9971	0.9900	0.9987	0.9963	0.9996	0.9984
LS&ICP	0.9512	0.6821	0.9692	0.8368	0.9837	0.9268	0.9942	0.9838
TPS&NICP	0.9876	0.9765	0.9962	0.9908	0.9989	0.9965	0.9998	0.9993

**Table 10:** The angles (°) between allometric vectors (permutation test *p* < 0.05 *) derived at varying densities.

	50	100	200	400	800
Sliding TPS LS&ICP	65.19 *	75.25 *	82.38 *	86.50 *	90.25 *
Sliding TPS TPS&NICP	6.52	7.67	8.68	8.97	8.73
LS&ICP TPS&NICP	65.83 *	75.68 *	82.82 *	86.19 *	90.16 *

**Table 11:** Procrustes distances between the predicted landmark and semilandmark configurations from sliding TPS and TPS&NICP corresponding to the maximum (Max) and minimum (Min) centroid sizes.

	50	100	200	400	800
Max	0.0095	0.0113	0.0122	0.0124	0.0134
Min	0.0168	0.0189	0.0211	0.0214	0.0202

## Data Availability

The data presented in this study are available on request from the corresponding author.

## Supplementary Materials

The following supporting information can be downloaded at: https://www.mdpi.com/article/10.3390/ani13071179/s1, Reference [53] is cited in the supplementary materials. Figure S1: Human head dataset: a human head surface with 16 anatomical landmarks; Figure S2: Locations of the average coordinates of 1000 semilandmarks generated by sliding TPS, LS&ICP, and TPS&NICP approaches; Figure S3: The average differences (Euclidean distance in location in mm) between 1000 semilandmarks generated by different approaches; Figure S4: The mean semilandmarks generated by sliding TPS over the face and scalp; Figure S5: Comparison of the centroid sizes of landmark and semilandmark configurations computed by different approaches; Figure S6: Vectors of Procrustes distances between each individual and the mean computed for each semilandmarking approach using different densities of semilandmarks; Figure S7: Comparison of the vector of Procrustes distances between every specimen and the mean among different approaches; Table S1: Comparison of semilandmarks from sliding TPS and TPS&NICP; Table S2: Comparison of semilandmarks from sliding TPS and LS&ICP; Table S3: Comparison of semilandmarks from TPS&NICP and LS&ICP; Table S4: The centroid sizes of the mean landmark and semilandmark configurations generated by different semilandmarking approaches and different densities of semilandmarks; Table S5: Procrustes distances computed between mean landmark and semilandmark configurations; Table S6: Procrustes distances computed between mean landmark and semilandmark configurations in the face and scalp; Table S7: Pearson correlations among vectors of Procrustes distances between each individual and the mean and Mantel tests of association between the Procrustes distance matrices derived using different semilandmarking approaches; Table S8: Pearson correlations among vectors of Procrustes distances between each individual and the mean and Mantel tests comparing Procrustes distance matrices between each density of semilandmarking and the maximum density; Table S9: Pearson and Mantel correlations between vectors and matrices of Procrustes distances from each semilandmarking approach and density and those from landmarks alone; Table S10: Pearson correlations between PC1 and PC2 scores derived using different semilandmarking approaches; Table S11: Pearson correlations of PC1 and PC2 scores between each semilandmark density and the maximum density; Table S12: Pearson correlations of PC1 and PC2 scores from landmarks alone and those from each semilandmarking approach and density; Table S13: Pearson correlations between PC1 and PC2 scores based on facial landmarks and semilandmarks derived using different semilandmarking approaches; Table S14: Correlations of PC1 and PC2 scores between each facial landmark and semilandmark configuration at lower densities of semilandmarks and the configuration with the maximum density; Table S15: Correlations of PC1 and PC2 scores derived from the landmarks alone and each facial landmark and semilandmark configuration generated by different approaches and densities; Table S16: The angles between allometric vectors from different semilandmarking approaches and densities; Table S17: Comparison of Procrustes distances between the predicted shapes corresponding to the maximum and minimum centroid size derived using semilandmarking approaches and densities.

## References

[1] O’Higgins P. (2000). The study of morphological variation in the hominid fossil record: Biology, landmarks and geometry. J. Anat..

[2] Adams D., Rohlf J., Slice D. (2004). Geometric morphometrics: Ten years of progress following the ‘revolution’. Ital. J. Zool..

[3] Viscosi V., Cardini A. (2011). Leaf morphology, taxonomy and geometric morphometrics: A simplified protocol for beginners. PLoS ONE.

[4] Mitteroecker P., Gunz P. (2009). Advances in geometric morphometrics. Evol. Biol..

[5] Mitteroecker P., Schaefer K. (2022). Thirty years of geometric morphometrics: Achievements, challenges, and the ongoing quest for biological meaningfulness. Am. J. Biol. Anthropol..

[6] Marshall A.F., Bardua C., Gower D.J., Wilkinson M., Sherratt E., Goswami A. (2019). High-density three-dimensional morphometric analyses support conserved static (intraspecific) modularity in caecilian (Amphibia: Gymnophiona) crania. Biol. J. Linn. Soc..

[7] Blanz V., Vetter T. A morphable model for the synthesis of 3D faces. Proceedings of the 26th Annual Conference on Computer Graphics and Interactive Techniques.

[8] Bookstein F.L. (1997). Landmark methods for forms without landmarks: Morphometrics of group differences in outline shape. Med. Image Anal..

[9] Oxnard C., O’Higgins P. (2009). Biology clearly needs morphometrics. Does morphometrics need biology?. Biol. Theory.

[10] Rolfe S., Davis C., Maga A.M. (2021). Comparing semi-landmarking approaches for analyzing three-dimensional cranial morphology. Am. J. Phys. Anthropol..

[11] Gunz P., Mitteroecker P., Bookstein F.L. (2005). Semilandmarks in three dimensions. Modern Morphometrics in Physical Anthropology.

[12] Perez S.I., Bernal V., Gonzalez P.N. (2006). Differences between sliding semi-landmark methods in geometric morphometrics, with an application to human craniofacial and dental variation. J. Anat..

[13] Gunz P., Mitteroecker P. (2013). Semilandmarks: A method for quantifying curves and surfaces. Hystrix Ital. J. Mammal..

[14] Chui H., Rangarajan A. (2003). A new point matching algorithm for non-rigid registration. Comput. Vis. Image Underst..

[15] Mydlová M., Dupej J., Koudelová J., Velemínská J. (2015). Sexual dimorphism of facial appearance in ageing human adults: A cross-sectional study. Forensic Sci. Int..

[16] Musilová B., Dupej J., Velemínská J., Chaumoitre K., Bruzek J. (2016). Exocranial surfaces for sex assessment of the human cranium. Forensic Sci. Int..

[17] Amberg B., Romdhani S., Vetter T. Optimal step nonrigid ICP algorithms for surface registration. Proceedings of the IEEE Conference on Computer Vision and Pattern Recognition (CVPR’07).

[18] Booth J., Roussos A., Ponniah A., Dunaway D., Zafeiriou S. (2018). Large scale 3D morphable models. Int. J. Comput. Vis..

[19] Shui W., Zhou M., Maddock S., Ji Y., Deng Q., Li K., Fan Y., Li Y., Wu X.J. (2020). A computerized craniofacial reconstruction method for an unidentified skull based on statistical shape models. Multimed. Tools Appl..

[20] White J.D., Ortega-Castrillón A., Matthews H., Zaidi A.A., Ekrami O., Snyders J., Fan Y., Penington T., Van Dongen S., Shriver M.D. (2019). MeshMonk: Open-source large-scale intensive 3D phenotyping. Sci. Rep..

[21] Dai H., Pears N., Smith W., Duncan C. (2020). Statistical Modeling of Craniofacial Shape and Texture. Int. J. Comput. Vis..

[22] Van Kaick O., Zhang H., Hamarneh G., Cohen-Or D. (2011). A survey on shape correspondence. Comput. Graph. Forum.

[23] Besl P.J., McKay N.D. (1992). A method for registration of 3-D shapes. IEEE Trans. Pattern Anal. Mach. Intell..

[24] Rusinkiewicz S., Levoy M. Efficient variants of the ICP algorithm. Proceedings of the Third IEEE International Conference on 3-D Digital Imaging and Modeling.

[25] Pomidor B.J., Makedonska J., Slice D.E. (2016). A landmark-free method for three-dimensional shape analysis. PLoS ONE.

[26] Boyer D.M., Puente J., Gladman J.T., Glynn C., Mukherjee S., Yapuncich G.S., Daubechies I. (2015). A new fully automated approach for aligning and comparing shapes. Anat. Rec..

[27] Vitek N.S., Manz C.L., Gao T., Bloch J.I., Strait S.G., Boyer D.M. (2017). Semi-supervised determination of pseudocryptic morphotypes using observer-free characterizations of anatomical alignment and shape. Ecol. Evol..

[28] Gao T., Yapuncich G.S., Daubechies I., Mukherjee S., Boyer D.M. (2018). Development and assessment of fully automated and globally transitive geometric morphometric methods, with application to a biological comparative dataset with high interspecific variation. Anat. Rec..

[29] Wang S., Wang Y., Jin M., Gu X.D., Samaras D. (2007). Conformal geometry and its applications on 3D shape matching, recognition, and stitching. IEEE Trans. Pattern Anal. Mach. Intell..

[30] Gu X., Wang Y., Chan T.F., Thompson P.M., Yau S.-T. (2004). Genus zero surface conformal mapping and its application to brain surface mapping. IEEE Trans. Med. Imaging.

[31] Boyer D.M., Lipman Y., Clair E.S., Puente J., Patel B.A., Funkhouser T., Jernvall J., Daubechies I. (2011). Algorithms to automatically quantify the geometric similarity of anatomical surfaces. Proc. Natl. Acad. Sci. USA.

[32] Koehl P., Hass J. (2015). Landmark-free geometric methods in biological shape analysis. J. R. Soc. Interface.

[33] Toussaint N., Redhead Y., Vidal-García M., Lo Vercio L., Liu W., Fisher E.M., Hallgrímsson B., Tybulewicz V.L., Schnabel J.A., Green J.B.J. (2021). A landmark-free morphometrics pipeline for high-resolution phenotyping: Application to a mouse model of Down syndrome. Development.

[34] Porto A., Rolfe S., Maga A.M. (2021). ALPACA: A fast and accurate computer vision approach for automated landmarking of three-dimensional biological structures. Methods Ecol. Evol..

[35] Gonzalez P.N., Barbeito-Andrés J., D’Addona L.A., Bernal V., Perez S.I. (2016). Performance of semi and fully automated approaches for registration of 3D surface coordinates in geometric morphometric studies. Am. J. Phys. Anthropol..

[36] Harper C.M., Goldstein D.M., Sylvester A.D. (2022). Comparing and combining sliding semilandmarks and weighted spherical harmonics for shape analysis. J. Anat..

[37] Profico A., Piras P., Buzi C., Di Vincenzo F., Lattarini F., Melchionna M., Veneziano A., Raia P., Manzi G. (2017). The evolution of cranial base and face in Cercopithecoidea and Hominoidea: Modularity and morphological integration. Am. J. Primatol..

[38] Schlager S. (2017). Morpho and Rvcg–Shape Analysis in R: R-Packages for geometric morphometrics, shape analysis and surface manipulations. Statistical Shape and Deformation Analysis.

[39] Shui W., Zhang Y., Wu X., Zhou M. (2021). A computerized facial approximation method for archaic humans based on dense facial soft tissue thickness depths. Archaeol. Anthropol. Sci..

[40] Dutilleul P., Stockwell J.D., Frigon D., Legendre P. (2000). The Mantel test versus Pearson’s correlation analysis: Assessment of the differences for biological and environmental studies. J. Agric. Biol. Environ. Stat..

[41] Klingenberg C.P. (2016). Size, shape, and form: Concepts of allometry in geometric morphometrics. Dev. Genes Evol..

[42] Gonzalez P.N., Perez S.I., Bernal V. (2010). Ontogeny of robusticity of craniofacial traits in modern humans: A study of South American populations. Am. J. Phys. Anthropol..

[43] Bastir M., García-Martínez D., Torres-Tamayo N., Palancar C.A., Fernández-Pérez F.J., Riesco-López A., Osborne-Márquez P., Ávila M., López-Gallo P. (2019). Workflows in a Virtual Morphology Lab: 3D scanning, measuring, and printing. J. Anthropol. Sci..

[44] Cardini A., O’Higgins P., Rohlf F.J. (2019). Seeing distinct groups where there are none: Spurious patterns from between-group PCA. Evol. Biol..

[45] Cardini A. (2020). Less tautology, more biology? A comment on “high-density” morphometrics. Zoomorphology.

[46] Schlager S., Rüdell A. (2017). Sexual Dimorphism and population affinity in the human zygomatic structure—Comparing surface to outline data. Anat. Rec..

[47] Shui W., Zhou M., Maddock S., He T., Wang X., Deng Q. (2017). A PCA-Based method for determining craniofacial relationship and sexual dimorphism of facial shapes. Comput. Biol. Med..

[48] Klingenberg C.P. (2013). Visualizations in geometric morphometrics: How to read and how to make graphs showing shape changes. Hystrix Ital. J. Mammal..

[49] Duncan C., Pears N.E., Dai H., Smith W.A.P., O’Higgins P. (2022). Applications of 3D Photography in Craniofacial Surgery. J. Paediatr. Neurosci..

[50] O’Higgins P., Cobb S.N., Fitton L.C., Gröning F., Phillips R., Liu J., Fagan M.J. (2011). Combining geometric morphometrics and functional simulation: An emerging toolkit for virtual functional analyses. J. Anat..

[51] O’Higgins P., Fitton L.C., Godinho R.M. (2019). Geometric morphometrics and finite elements analysis: Assessing the functional implications of differences in craniofacial form in the hominin fossil record. J. Archaeol. Sci..

[52] Shui W., Profico A., O’Higgins P. (2023). A comparison of semilandmarking approaches in the visualisation of shape differences. Animals.

[53] Smith O.A., Nashed Y.S., Duncan C., Pears N., Profico A., O’Higgins P. (2020). 3D Modeling of craniofacial ontogeny and sexual dimorphism in children. Anat. Rec..

