# Supplementary material for: A Comparison of Semilandmarking Approaches in the Analysis of Size and Shape
Source: Animals (Basel). 2023 Mar 28;13(7):1179. doi: 10.3390/ani13071179 (PMC10093231; doi:10.3390/ani13071179)
Supplement: Supplementary file 1 [file animals-13-01179-s001.zip › animals-2189614-supplementary.pdf]

Supplementary materials

A Comparison of Semilandmarking Approaches in the Analysis of Size and Shape

Wuyang Shui [1,*], Antonio Profico [2] and Paul O'Higgins [1,3]

[1] Department of Archaeology, University of York, York YO1 7EP, UK
[2] Department of Biology, University of Pisa, via Derna 1, Pisa 56126, Italy
[3] Hull York Medical School, University of York, York YO10 5DD, UK

Analyses of human head surface data

1. Material

Adult Human Heads

The sample comprises 100 surface scans of adult human male heads from the Liverpool–York 3D 'headspace' dataset. Each human head surface scan comprises more than 146,000 vertices and 290,000 triangles. Geometry and texture were captured using a 3dMD five-camera system [21]. Further, 16 anatomical (homologous) landmarks had already been semiautomatically marked up on each, in the course of a previous study [53], and these are shown in Figure S1. These cover much of the facial region, but landmarks are not identifiable over the scalp.

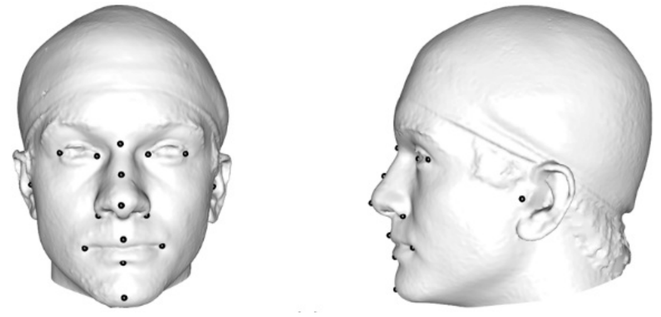

Figure S1. Human head dataset: a human head surface with 16 anatomical landmarks

2. Methods

2.1. Generation of the template

A landmark and semilandmark template was created to be used as the basis of semilandmarking using three different approaches. To create this template, we used NICP [17] to align all of the human heads using landmarks as a 'soft constraint', and from this alignment, we computed a mean template form (size and shape) surface by averaging the coordinates of every vertex of all the heads. Then, we used the k-means clustering algorithm to evenly distribute five different densities (200, 400, 600, 800, and 1000) of surface semilandmarks over the template, ignoring the locations of true landmarks and avoiding the ears, which have a complex surface.

2.2 Semilandmarking approaches

Once the template was created, the three methods of semilandmarking described above were applied as described in the main paper.

2.3 Comparison of three semilandmarking approaches

As with the ape cranial surfaces, we compared the different semilandmarking approaches by testing the null hypotheses described in the Introduction. These relate to differences in locations of semilandmarks, estimates of mean sizes and shapes, patterns of variation, and covariation of shape with size (allometry).

3. Results

3.1. The locations of semilandmarks

The resulting differences in placement of semilandmarks ('errors') from the three semilandmarking approaches are presented in Tables S1-3 for the comparisons of sliding TPS and TPS&NICP, sliding TPS and LS&ICP, and TPS&NICP and LS&ICP approaches, respectively. The tables list differences in location (diff, in mm), the average deviation (dev in mm) and the % of semilandmarks that differ in location by 0.0-1.0 mm, 1-2.5 mm, 2.5-5 mm, and ≥5.0 mm.

Table S1. Comparison of semilandmarks from sliding TPS and TPS&NICP.

diff mm	200		400		600		800		1000	
	dev	%	dev	%	dev	%	dev	%	dev	%
[0.0-1.0)	0.68	16.00	0.71	15.75	0.72	15.17	0.74	15.13	0.75	15.00
[1.0-2.5)	1.63	18.00	1.68	21.75	1.69	23.67	1.68	27.12	1.72	29.40
[2.5-5.0)	3.81	40.00	3.83	45.25	3.74	50.67	3.73	52.25	3.67	55.50
≥5.00	5.75	26.00	5.64	17.25	5.35	10.50	5.12	5.50	5.02	0.10
Total	3.42	100.00	3.18	100.00	2.96	100.00	2.80	100.00	2.66	100.00

Table S2. Comparison of semilandmarks from sliding TPS and LS&ICP.

diff. mm	200		400		600		800		1000	
	dev	%	dev	%	dev	%	dev	%	dev	%
[0.0-1.0)	-	-	-	-	-	-	-	-	-	-
[1.0-2.5)	-	-	-	-	-	-	-	-	-	-
[2.5-5.0)	4.21	37.50	4.26	47.25	4.28	51.50	4.34	58.63	4.36	52.60
≥5.00	5.83	62.50	5.60	52.75	5.54	48.50	5.51	41.37	5.52	47.40
Total	5.22	100.00	4.97	100.00	4.89	100.00	4.82	100.00	4.91	100.00

Table S3. Comparison of semilandmarks from TPS&NICP and LS&ICP.

diff. mm	200		400		600		800		1000	
	dev	%	dev	%	dev	%	dev	%	dev	%
[0.0-1.0)	-	-	-	-	-	-	-	-	-	-
[1.0-2.5)	-	-	-	-	-	-	-	-	-	-
[2.5-5.0)	4.35	12.50	4.37	15.00	4.33	15.83	4.38	20.25	4.38	17.90
≥5.00	6.84	87.50	6.56	85.00	6.45	84.17	6.43	79.75	6.40	82.10
Total	6.53	100.00	6.23	100.00	6.12	100.00	6.02	100.00	6.04	100.00

The mean locations of 1000 semilandmarks generated by sliding TPS (black points), LS&ICP (amber points), and TPS&NICP (magenta points) approaches are illustrated in Figure S2 on the template surface warped to the mean of the sliding TPS generated landmark and semilandmarks. It is evident that their locations differ among semilandmarking approaches. The mean configurations from both TPS&NICP and LS&ICP lie close to, or on, the surface, defined by the mean configuration from sliding TPS. Similar results are found for all semilandmarking densities, with sliding TPS and TPS&NICP approaches producing the most similar semilandmark locations.

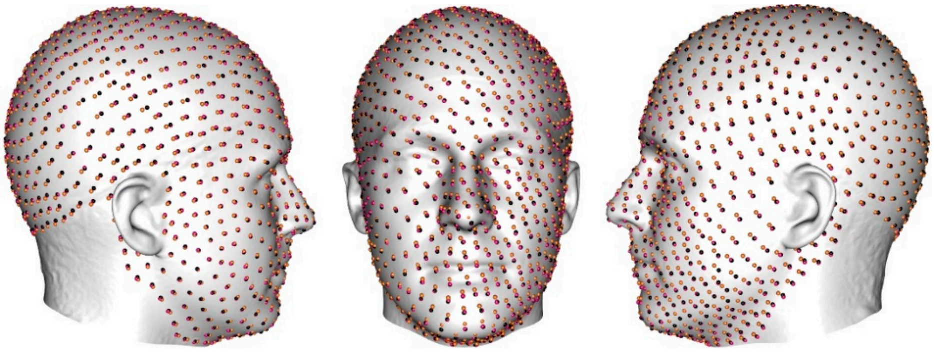

Figure S2. Locations of the average coordinates of 1000 semilandmarks generated by sliding TPS (black points), LS&ICP (amber points), and TPS&NICP (magenta points) approaches.

Focusing on the highest semilandmarking density (but note that these findings apply equally to all higher densities), Figure S3a shows the differences in location among 1000 semilandmarks generated by sliding TPS and TPS&NICP approaches. Further, 15% (Table S1) of the full set of semilandmarks (amber points) differ in location by <1.0 mm and nearly all of these are located on the face (Figure S3a), where the density of landmarks is high. As the density of landmarks in a region falls, the differences in semilandmark placement increase. Thus, 29.4% (Table S1) of the semilandmarks (Figure S3a, blue points) present a difference of 1.0-2.5 mm, principally around the forehead and cheeks, and 55.6% of the semilandmarks (magenta and black points) present differences greater than 2.5 mm in location among semilandmarking approaches; principally, these larger differences are located over the scalp. Figure S3b shows the differences in placement of 1000 semilandmarks generated by sliding TPS and LS&ICP approaches. Compared to Figure S3a, the deviations between equivalent semilandmarks are larger. This reflects the differences presented in Table S2. For 1000 semilandmarks, all deviations between sliding TPS and LS&ICP are >2.5mm, while sliding TPS and TPS&NICP place 44.4% of semilandmarks with a difference in location of less than 2.5 mm. These greater differences are most evident in the face (Figure S3b) where increased asymmetry of differences in the locations of semilandmarks is also evident.

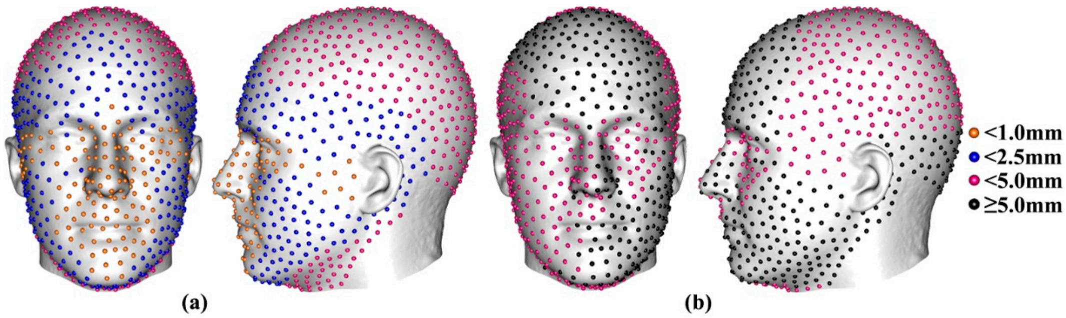

Figure S3. The average differences (Euclidean distance in location in mm) between 1000 semilandmarks generated by different approaches. (a) Differences between sliding TPS and TPS&NICP. (b) Differences between sliding TPS and LS&ICP. Differences between TPS&NICP and LS&ICP approaches are not shown because they are very similar to those in b.

3.2. Comparison of landmark and semilandmark configurations among different semilandmarking approaches

For adult human male heads, the centroid sizes of the estimated mean configurations derived from different semilandmarking approaches are presented in Table S4. They indicate that the mean centroid sizes estimated using each approach are almost the same and increase similarly with increasing semilandmark density, as expected.

Table S4. The centroid sizes of the mean landmark and semilandmark configurations generated by different semilandmarking approaches and different densities of semilandmarks.

	**200**	**400**	**600**	**800**	**1000**
Sliding TPS	1514	2126	2599	2990	3334
LS&ICP	1513	2123	2595	2986	3330
TPS&NICP	1513	2124	2598	2989	3333

Additionally, Procrustes distances were computed among estimates of the mean landmark and semilandmark configurations from different semilandmarking approaches (Table S5). Comparison of these distances among semilandmarking methods and densities indicates that the estimates of the mean landmark and semilandmark configurations generated from sliding TPS are more similar to TPS&NICP than to LS&ICP. With increasing numbers of semilandmarks, the LS&ICP approach converges on the results obtained using sliding TPS and TPS&NICP, as indicated by the progressively reducing Procrustes distances with increasing semilandmarking density in rows 1 and 3 of Table S5. In contrast, the estimated mean configurations from sliding TPS and TPS&NICP present a very similar Procrustes distance (0.0049+/- 1) at all densities.

Table S5. Procrustes distances (permutation test $p < 0.05$ *) computed between mean landmark and semilandmark configurations.

	200	400	600	800	1000
Sliding TPS LS&ICP	0.0112*	0.0093*	0.0086*	0.0083*	0.0082*
Sliding TPS TPS&NICP	0.0048	0.0049	0.0049	0.0050	0.0049
LS&ICP TPS&NICP	0.0122*	0.0103*	0.0096*	0.0093*	0.0091*

At each semilandmarking density, the estimates of mean shape arising from the different semilandmarking approaches differ by between 14.15% and 37.43% of the average difference between individuals and the mean, estimated using sliding TPS semilandmarking. Between sliding TPS and TPS&NICP, this relative distance remains stable (~14.5%) with increasing semilandmark density, whereas it decreases from ~35% to 25% for the comparisons between the mean derived by LS&ICP and those from both sliding TPS and TPS&NICP.

To further explore why estimates of mean landmark and semilandmark configurations from different semilandmarking approaches show the patterns of difference presented in Table S5, the semilandmarks generated by sliding TPS were separated into two regions, 'face' (amber points) and 'scalp' (magenta points), as in Figure S4. Then, Procrustes distances were computed between the landmark and semilandmark sets from these regions, obtained using different approaches and semilandmarking densities (Table S6). At all densities, the Procrustes distances between landmarks and semilandmarks in the face and scalp, computed from sliding TPS and TPS&NICP, are the smallest, with distances among face semilandmarks being the largest and these are consistent among semilandmarking densities. This contrasts with the differences in semilandmark locations (Figures S2 and S3), which are greater in the scalp than face, because the rotation step of Procrustes reduces the systematic errors in scalp landmarks evident in Figure S2. The Procrustes distances from LS&ICP and both sliding TPS and TPS&NICP (Table S6 rows 1 and 3) are larger. Similar to the comparison of sliding TPS and TPS&NICP, distances are greater among facial landmarks and semilandmarks than among scalp semilandmarks. Further, the distances among facial landmarks and semilandmarks from LS&ICP and both sliding TPS and TPS&NICP decrease with increasing semilandmark density. This likely accounts for the convergence with increasing numbers of semilandmarks between the LS&ICP approach and both sliding TPS and TPS&NICP mean shapes seen in Table S5.

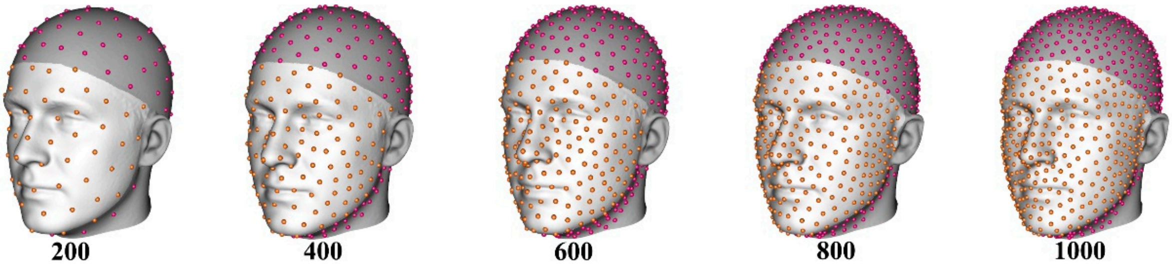

Figure S4. The mean semilandmarks generated by sliding TPS over the face (amber points) and scalp (magenta points).

Table S6. Procrustes distances (permutation test $p < 0.05$ *, $p < 0.1$ #) computed between mean landmark and semilandmark configurations in the face and scalp.

	200		400		600		800		1000	
	**Face**	**Scalp**	**Face**	**Scalp**	**Face**	**Scalp**	**Face**	**Scalp**	**Face**	**Scalp**
Sliding TPS LS&ICP	0.0229*	0.0067*	0.0195*	0.0058*	0.0179*	0.0058*	0.0168*	0.0060*	0.0166*	0.0062*
Sliding TPS TPS&NICP	0.0056	0.0055	0.0057	0.0055	0.0061	0.0054	0.0061	0.0056	0.0061	0.0054
LS&ICP TPS&NICP	0.0238*	0.0081*	0.0205*	0.0074#	0.0190*	0.0072#	0.0181*	0.0071#	0.0175*	0.0072#

3.3. Comparison of centroid sizes and Procrustes distance matrices

3.3.1. Differences between semilandmarking approaches

The centroid sizes of individuals, computed using the landmarks and semilandmarks generated by alternative semilandmarking approaches, are compared between sliding TPS and the other two semilandmarking approaches in Figure S5 (the comparison of TPS&NICP and LS&ICP approaches is not shown because it is very similar to those in Figure S5a). Figure S5a compares centroid sizes between sliding TPS (horizontal axis) and LS&ICP (vertical axis). The dashed line denotes the expected relationship if centroid sizes are identical, and the red line represents the fitted line from a regression of centroid sizes of individuals from the landmark and semilandmark configurations computed by LS&ICP (dependent variable) on those computed by sliding TPS (independent variable). In each case, the fitted red line shows a smaller gradient than the expected linear relationship indicated by the dashed line. Likewise, Figure S5b compares centroid sizes from sliding TPS (independent variable) and TPS&NICP (dependent variable). The plots indicate that the centroid sizes from sliding TPS (red lines) were very similar to those of TPS&NICP at all assessed semilandmarking densities.

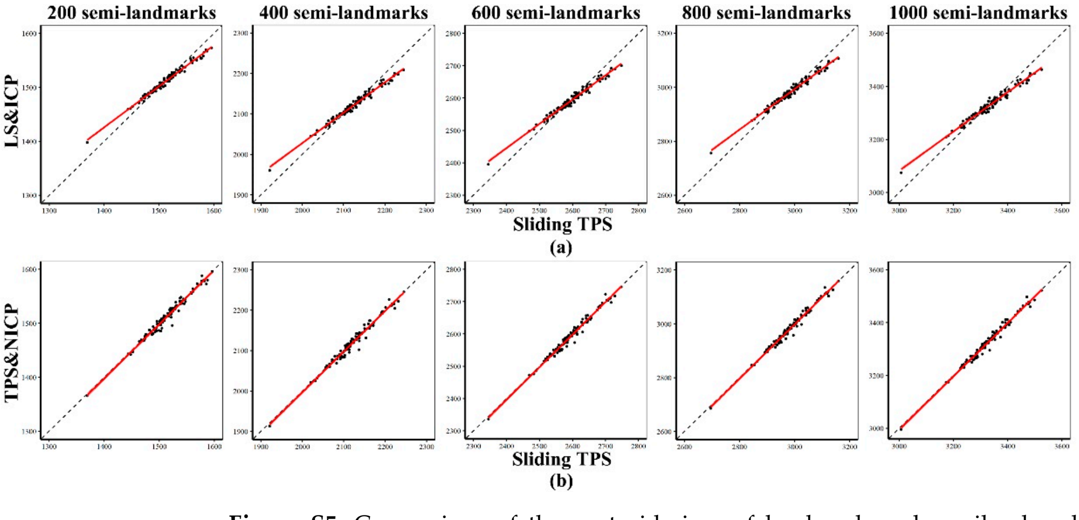

Figure S5. Comparison of the centroid sizes of landmark and semilandmark configurations computed by different approaches. (a) Comparison of TPS sliding and LS&ICP. (b) Comparison of TPS sliding and TPS&NICP. TPS&NICP and LS&ICP approaches are not compared in this figure because the results are very similar to those in a.

Figure S6 summarizes the vectors of Procrustes distances between each individual and the mean, calculated using landmarks and

semilandmarks from the sliding TPS, LS&ICP, and TPS&NICP approaches. In Figure S6, the horizontal axis represents different densities of semilandmarks and the vertical axis represents Procrustes distances. Consistently across semilandmarking densities within each method, the smallest distances arise from the LS&ICP approach and the largest from TPS&NICP, with sliding TPS intermediate.

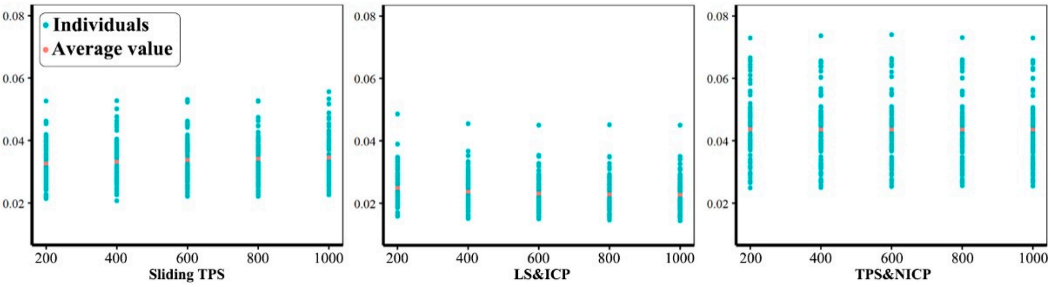

Figure S6. Vectors of Procrustes distances between each individual and the mean computed for each semilandmarking approach using different densities of semilandmarks. Cyan points represent Procrustes distance between every specimen and the Procrustes mean shape and red points represent the average value of the Procrustes distance vector.

Figure S7 directly compares the distances from the sliding TPS approach with those from the other two approaches. The results comparing TPS&NICP and LS&ICP are not shown, because they are similar to those in Figure S7a comparing vectors of Procrustes distances between each individual and the mean produced by sliding TPS (horizontal axis) and LS&ICP (vertical axis). The dashed line denotes the expected relationship if Procrustes distance vectors are identical, and the red line represents the linearly fitted regression of the vector derived from LS&ICP (dependent variable) on the vector from sliding TPS (independent variable). For each density of semilandmarking, the fitted red line has a smaller gradient and lies below the expected linear relationship indicated by the dashed line, indicating that Procrustes distances from sliding TPS are greater than those from LS&ICP (supported by Figure S6) and this difference increases as Procrustes distance increases. Likewise, Figure S7b compares Procrustes distance vectors between sliding TPS and TPS&NICP. The plots indicate that the Procrustes distance vectors from sliding TPS are less than those from TPS&NICP at all semilandmarking densities.

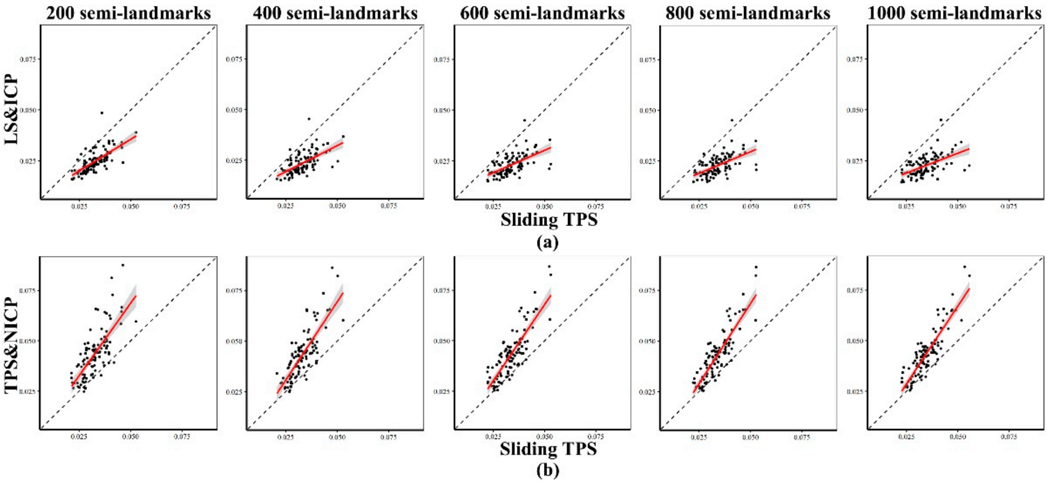

Figure S7. Comparison of the vector of Procrustes distances between every specimen and the mean among different approaches. (a) Comparison of TPS sliding and LS&ICP approaches. (b) Comparison of TPS sliding and TPS&NICP approaches. TPS&NICP and LS&ICP approaches are not compared in this figure because the results are similar to those in a.

Table S7 compares the Pearson correlations among the vectors of Procrustes distances between every individual and the mean as well as the Mantel correlation between the Procrustes distance matrices. At all semilandmarking densities, the largest Pearson correlations (*r*) are between Procrustes distance vectors from sliding TPS and TPS&NICP, and these correlations increase with increasing numbers of semilandmarks (second row in Table S7). In contrast, the weakest correlations are consistently between LS&ICP and TPS&NICP and these decrease with increasing semilandmark density.

Using Mantel tests, Table S7 also compares the matrices of Procrustes distances among all individuals calculated using landmarks and semilandmarks generated by the different semilandmarking approaches. The largest matrix correlations were found between sliding TPS and TPS&NICP, and the association becomes stronger with increasing numbers of semilandmarks (second row in Table S7). The weakest association, as assessed by Mantel tests, is between matrices calculated from semilandmarks derived from LS&ICP and TPS&NICP. These become weaker with increasing numbers of semilandmarks. Additionally, the matrix (Mantel) correlations for this comparison are consistently low relative to the other comparisons (bottom row in Table S7 vs. rows 1 and 2).

Table S7. Pearson correlations (all $p < 0.001$) among vectors of Procrustes distances between each individual and the mean and Mantel tests (all $p < 0.001$) of association between the Procrustes distance matrices derived using different semilandmarking approaches**.**

	**200**		**400**		**600**		**800**		**1000**	
	**Pearson**	**Mantel**	**Pearson**	**Mantel**	**Pearson**	**Mantel**	**Pearson**	**Mantel**	**Pearson**	**Mantel**
Sliding TPS LS&ICP	0.6830	0.6841	0.6068	0.6260	0.5790	0.5978	0.5555	0.5790	0.5323	0.5528
Sliding TPS TPS&NICP	0.7284	0.7017	0.7901	0.7576	0.8304	0.8009	0.8623	0.8311	0.8829	0.8564
LS&ICP TPS&NICP	0.3955	0.3916	0.3615	0.3646	0.3478	0.3504	0.3438	0.3454	0.3368	0.3382

3.3.2. Different densities of semilandmarks

Consistency within semilandmarking methods, of relative Procrustes distances among specimens computed using varying densities of semilandmarks, was assessed by tests of association. Pearson correlations were computed among vectors of Procrustes distances between each individual and the mean, as were Mantel correlations among the matrices of Procrustes distances. These, presented in Table S8, compared the distances from the landmark and semilandmark configuration comprising 1000 semilandmarks from the head surface data with those from configurations comprising 200-800 semilandmarks. Within each semilandmarking approach, these correlations are generally large (>0.90) and increase with increasing numbers of semilandmarks. The largest correlations are found across semilandmarking densities

arising from TPS&NICP and the smallest, from sliding TPS, especially at lower semilandmarking densities.

Table S8. Pearson correlations (all $p < 0.001$) among vectors of Procrustes distances between each individual and the mean and Mantel tests comparing Procrustes distance matrices between each density of semilandmarking and the maximum density.

	200		400		600		800	
	**Pearson**	**Mantel**	**Pearson**	**Mantel**	**Pearson**	**Mantel**	**Pearson**	**Mantel**
Sliding TPS	0.9029	0.9075	0.9580	0.9597	0.9829	0.9832	0.9886	0.9898
LS&ICP	0.9919	0.9893	0.9975	0.9969	0.9981	0.9977	0.9991	0.9990
TPS&NICP	0.9978	0.9976	0.9994	0.9993	0.9994	0.9994	0.9998	0.9997

Additionally, the Pearson correlations among vectors of Procrustes distances between each individual and the mean and the Mantel correlations among distance matrices were computed between distances derived from each semilandmarking approach and density and the distances based on landmarks alone are computed (Table S9). The TPS&NICP approach results in distances that are most highly correlated with those from landmarks and the sliding TPS approach results in distances that are a little less strongly correlated, while the LS&ICP approach results in distances that are poorly correlated with those from landmarks alone. For LS&ICP and TPS&NICP approaches, but not sliding TPS, the correlations become smaller with increasing semilandmarking density.

Table S9. Pearson and Mantel correlations (all $p < 0.01$, except $^{\#}$, $p < 0.05$) between vectors and matrices of Procrustes distances from each semilandmarking approach and density and those from landmarks alone.

	200		400		600		800		1000	
	**Pearson**	**Mantel**	**Pearson**	**Mantel**	**Pearson**	**Mantel**	**Pearson**	**Mantel**	**Pearson**	**Mantel**
Sliding TPS	0.6424	0.5718	0.6604	0.5826	0.6729	0.5910	0.6928	0.6084	0.6848	0.6115
LS&ICP	0.3234	0.3164	0.2677	0.2634	0.2549$^{\#}$	0.2472	0.2469$^{\#}$	0.2378	0.2342$^{\#}$	0.2260
TPS&NICP	0.7315	0.7035	0.7237	0.6976	0.7221	0.6949	0.7222	0.6952	0.7208	0.6944

3.4. PCA and allometry

3.4.1. Correlations between PC scores from different semilandmarking methods and densities

To broadly compare distributions of specimens arising from different semilandmarking approaches and densities, correlations (ignoring sign, and so arbitrary reflections on PCs) were computed between scores of individuals on the first two PCs of shape derived from separate GPA/PCA of the landmark and semilandmark configurations (Table S10). These first two PCs account for greater than 30% of the total shape variance in each analysis of landmarks and semilandmarks from sliding TPS and LS&ICP, and 49% for those from TPS&NICP. Correlations are generally moderate to weak. They are low for comparisons of PC1 and PC2 scores between LS&ICP and TPS&NICP and become smaller for comparisons of PC1 scores between LS&ICP and sliding TPS with increasing density. In contrast, PC1 and PC2 correlations from sliding TPS and TPS&NICP are low for lower densities, rising for higher ones.

Table S10. Pearson correlations (all $p < 0.01$, except $^{\#}$, $p < 0.05$, *= n.s.) between PC1 and PC2 scores derived using different semilandmarking approaches.

	200		400		600		800		1000	
	**PC1**	**PC2**	**PC1**	**PC2**	**PC1**	**PC2**	**PC1**	**PC2**	**PC1**	**PC2**
Sliding TPS<br>LS&ICP	0.6246	0.0788*	0.5441	0.6581	0.4280	0.8798	0.3951	0.7776	0.3804	0.5059
Sliding TPS<br>TPS&NICP	0.2829	0.4818	0.4947	0.0921*	0.7287	0.2434$^{\#}$	0.8004	0.5523	0.7954	0.6903
LS&ICP<br>TPS&NICP	0.1436*	0.1030*	0.1621*	0.0848*	0.1727*	0.0713*	0.1718*	0.0411*	0.1735*	0.0085*

Within each semilandmarking approach, the correlations among PC1 and PC2 scores from every density and the maximum density of each semilandmarking approach are presented in Table S11. The correlations from LS&ICP and TPS&NICP were consistently large, whereas the correlations from sliding TPS increase with increasing semilandmark densities. Table S12 details the extent to which PC scores for the analyses in Table S11 correlate with those from PCA of landmarks alone. For TPS&NICP landmarks and semilandmarks, at all densities, PC1 scores show a consistent correlation of approximately 0.89, and PC2 scores, ~0.49. In contrast, PC scores based on landmarks and semilandmarks from sliding TPS show a lower correlation for PC1~0.55, except at the lowest density of semilandmarking where the correlation is 0.2437. PC2 correlations are uniformly low. LS&ICP semilandmarking at all densities results in PC1 and PC2 scores with uniformly low correlations.

Table S11. Pearson correlations (all $p < 0.001$) of PC1 and PC2 scores between each semilandmark density and the maximum (1000) density.

	200		400		600		800	
	**PC1**	**PC2**	**PC1**	**PC2**	**PC1**	**PC2**	**PC1**	**PC2**
Sliding TPS	0.4697	0.5826	0.7051	0.4900	0.9532	0.6054	0.9815	0.9131
LS&ICP	0.8661	0.8934	0.9469	0.9534	0.9799	0.9805	0.9942	0.9938
TPS&NICP	0.9989	0.9988	0.9994	0.9992	0.9996	0.9995	0.9998	0.9998

Table S12. Pearson correlations (all $p < 0.01$, except $^{\#}$, $p < 0.05$, *= n.s.) of PC1 and PC2 scores from landmarks alone and those from each semilandmarking approach and density.

	200		400		600		800		1000	
	**PC1**	**PC2**	**PC1**	**PC2**	**PC1**	**PC2**	**PC1**	**PC2**	**PC1**	**PC2**
Sliding TPS	0.2437$^{\#}$	0.2256$^{\#}$	0.4119	0.0538*	0.5586	0.0485*	0.6165	0.1081*	0.5782	0.2153$^{\#}$
LS&ICP	0.0385*	0.1486*	0.0422*	0.1751*	0.0342*	0.1821*	0.0222*	0.1929*	0.0240*	0.2085$^{\#}$
TPS&NICP	0.8897	0.4997	0.8844	0.4937	0.8847	0.4913	0.8877	0.4906	0.8932	0.4931

To assess the extent to which scalp semilandmarks, which lack nearby fixed landmarks to guide their placement, affect the relative performance of semilandmarking approaches, a set of PCAs was carried out using facial landmarks and semilandmarks alone. Table S13 presents the resulting correlations among PC scores extracted using each semilandmarking approach at each density of semilandmarking. These first two PCs account for almost 40% of the total shape variance in each analysis. Those between sliding TPS and TPS&NICP are consistently high (>0.9), while correlations between LS&ICP and both sliding TPS and

TPS&NICP are consistently low. In Table S14, for each semilandmarking approach, the correlations are presented between scores on PC1 and PC2 calculated using maximum semilandmarks and each lower semilandmarking density on the face. These correlations are consistently high for the sliding TPS and TPS&NICP approaches, while they are consistently low for the LS&ICP approach. Table S15 presents the correlations between PC1 and PC2 scores computed using the facial landmarks alone and those from the set of landmarks and semilandmarks over the face derived from each semilandmarking approach and density. TPS&NICP-derived PC1 scores are consistently highly correlated with those from the landmarks, and PC2 scores are less strongly, but still highly, correlated. The PC scores from the sliding TPS approach show intermediate levels of correlation with those from landmarks, and the LS&ICP approach presents the lowest correlation.

Table S13. Pearson correlations (all $p < 0.01$, except #, $p < 0.05$, *= n.s.) between PC1 and PC2 scores based on facial landmarks and semilandmarks derived using different semilandmarking approaches.

	200		400		600		800		1000	
	**PC1**	**PC2**	**PC1**	**PC2**	**PC1**	**PC2**	**PC1**	**PC2**	**PC1**	**PC2**
Sliding TPS LS&ICP	0.2112#	0.5431	0.2130#	0.4862	0.2095#	0.4510	0.2245#	0.3525	0.2445#	0.2261#
Sliding TPS TPS&NICP	0.9617	0.9388	0.9520	0.9258	0.9525	0.9338	0.9603	0.9351	0.9654	0.9337
LS&ICP TPS&NICP	0.0859*	0.4849	0.0626*	0.4541	0.0565*	0.4235	0.0912*	0.3150	0.1310*	0.1869*

Table S14. Correlations (all $p < 0.001$) of PC1 and PC2 scores between each facial landmark and semilandmark configuration at lower densities of semilandmarks and the configuration with the maximum density.

	200		400		600		800	
	**PC1**	**PC2**	**PC1**	**PC2**	**PC1**	**PC2**	**PC1**	**PC2**
Sliding TPS	0.9861	0.9855	0.9941	0.9943	0.9944	0.9931	0.9982	0.9976
LS&ICP	0.8549	0.8663	0.9149	0.9196	0.9429	0.9447	0.9848	0.9846
TPS&NICP	0.9971	0.9915	0.9996	0.9991	0.9998	0.9994	0.9998	0.9993

Table S15. Correlations (all $p < 0.01$, except #, $p < 0.05$, *= n.s.) of PC1 and PC2 scores derived from the landmarks alone and each facial landmark and semilandmark configuration generated by different approaches and densities.

	200		400		600		800		1000	
	**PC1**	**PC2**	**PC1**	**PC2**	**PC1**	**PC2**	**PC1**	**PC2**	**PC1**	**PC2**
Sliding TPS	0.8371	0.5124	0.7841	0.3921	0.7775	0.4043	0.8021	0.3888	0.8159	0.4043
LS&ICP	0.1129*	0.2667	0.1775*	0.2187#	0.2052#	0.1938*	0.1921*	0.1522*	0.1755*	0.1097*
TPS&NICP	0.9193	0.7084	0.9023	0.6413	0.8955	0.6351	0.8997	0.6233	0.8971	0.6407

3.4.2. Comparison of allometric vectors

To assess the effects of the semilandmarking approach on allometric vectors at each density, the landmark and semilandmark configurations from each approach were submitted to a joint GPA followed by PCA. Angles, as a measure of dissimilarity, were then computed among allometric vectors estimated by multivariate regression of shape (scores on all PCs) on the natural logarithm of centroid size (Table S16). Angles

exceed 90° for some comparisons because the allometric vectors were compared respecting their polarity (i.e., from small to large). At all semilandmarking densities, the angles between sliding TPS and TPS&NICP derived vectors were smaller than those in comparisons involving LS&ICP. This indicates that the allometric vector resulting from semilandmarking using the LS&ICP approach is rather different from those derived from sliding TPS and TPS&NICP. Meanwhile, the angles between allometric vectors from sliding TPS and TPS&NICP become smaller with increasing semilandmark densities, whereas they increase in the comparisons with LS&ICP.

Table S16. The angles (°) between allometric vectors (permutation test $p < 0.05$*) from different semilandmarking approaches and densities.

	200	400	600	800	1000
Sliding TPS LS&ICP	87.39*	90.21*	92.37*	93.44*	93.68*
Sliding TPS TPS&NICP	32.47	29.57	26.29	24.00	23.12
LS&ICP TPS&NICP	92.76*	97.60*	98.64*	100.01*	100.32*

3.4.3. Comparison of allometric scaling of landmark and semilandmark configurations

We compared the predicted landmark and semilandmark configurations representing the extreme limits (maximum and minimum centroid sizes) of each allometric vector derived from each semilandmarking method by computing Procrustes distances between them. The results are presented in Table S17. The Procrustes distances among predictions are smallest and converge with increasing density for comparisons between predicted shapes from sliding TPS and TPS&NICP. For these comparisons, the distances among predicted shapes at minimum size (~0.024) are approximately double those at maximum size (~0.012) because the distribution of centroid sizes is skewed towards the maximum. These distances are approximately 70% of the average Procrustes distance to the mean for the comparisons of predicted shapes at minimum centroid size and 35% at maximum centroid size. Distances between predicted shapes from LS&ICP and those from the other semilandmarking approaches are generally greater.

Taken together, these findings indicate that predicted landmark and semilandmark configurations from allometric analysis are most similar between the sliding TPS and TPS&NICP approaches and least between LS&ICP and the other approaches.

Table S17. Comparison of Procrustes distances between the predicted shapes corresponding to the maximum (Max) and minimum (Min) centroid size derived using semilandmarking approaches and densities.

	200		400		600		800		1000	
	**Max**	**Min**	**Max**	**Min**	**Max**	**Min**	**Max**	**Min**	**Max**	**Min**
Sliding TPS LS&ICP	0.0298	0.0494	0.0288	0.0495	0.0281	0.0504	0.0278	0.0520	0.0283	0.0529
Sliding TPS TPS&NICP	0.0128	0.0260	0.0131	0.0257	0.0125	0.0231	0.0118	0.0222	0.0117	0.0217

LS&ICP TPS&NICP	0.0352	0.0628	0.0355	0.0653	0.0347	0.0646	0.0347	0.0659	0.0350	0.0668

4. Discussion

See main body of the paper.
